# Quantitative Insights into Phosphate-Enhanced Lead Immobilization on Goethite

**DOI:** 10.1021/acs.est.4c03927

**Published:** 2024-06-24

**Authors:** Wanli Lian, Guanghui Yu, Jie Ma, Juan Xiong, Cuiyun Niu, Ran Zhang, Haijiao Xie, Liping Weng

**Affiliations:** † Key Laboratory for Environmental Factors Control of Agro-Product Quality Safety, Agro-Environmental Protection Institute, Ministry of Agriculture and Rural Affairs, Tianjin 300191, China; ‡ Department of Soil Quality, Wageningen University, P.O. Box 47, 6700AA Wageningen, The Netherlands; § Institute of Surface-Earth System Science, School of Earth System Science, Tianjin University, Tianjin 300072, China; ∥ Key Laboratory of Arable Land Conservation (Middle and Lower Reaches of Yangtze River), Ministry of Agriculture and Rural Affairs of the People’s Republic of China, College of Resources and Environment, Huazhong Agricultural University, Wuhan 430070, China; ⊥ Hangzhou Yanqu Information Technology Co., Ltd, Hangzhou 310003, China

**Keywords:** lead, phosphate, ternary complex, precipitation, CD-MUSIC model, X-ray absorption fine structure, density functional theory

## Abstract

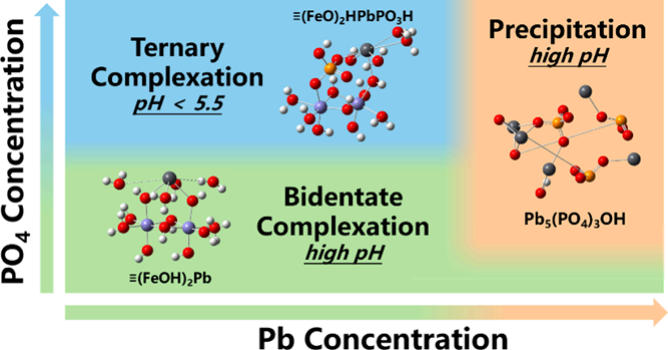

Despite extensive study, geochemical modeling often fails to accurately predict lead (Pb) immobilization in environmental samples. This study employs the Charge Distribution MUlti-SIte Complexation (CD-MUSIC) model, X-ray absorption fine structure (XAFS), and density functional theory (DFT) to investigate mechanisms of phosphate (PO_4_) induced Pb immobilization on metal (hydr)oxides. The results reveal that PO_4_ mainly enhances bidentate-adsorbed Pb on goethite via electrostatic synergy at low PO_4_ concentrations. At relatively low pH (below 5.5) and elevated PO_4_ concentrations, the formation of the monodentate-O-sharing Pb-PO_4_ ternary structure on goethite becomes important. Precipitation of hydropyromorphite (Pb_5_(PO_4_)_3_OH) occurs at high pH and high concentrations of Pb and PO_4_, with an optimized log *K*_sp_ value of −82.02. The adjustment of log *K*_sp_ compared to that in the bulk solution allows for quantification of the overall Pb-PO_4_ precipitation enhanced by goethite. The CD-MUSIC model parameters for both the bidentate Pb complex and the monodentate-O-sharing Pb-PO_4_ ternary complex were optimized. The modeling results and parameters are further validated and specified with XAFS analysis and DFT calculations. This study provides quantitative molecular-level insights into the contributions of electrostatic enhancement, ternary complexation, and precipitation to phosphate-induced Pb immobilization on oxides, which will be helpful in resolving controversies regarding Pb distribution in environmental samples.

## Abstract 2

This study provides comprehensive insights into mechanisms of lead immobilization on iron (hydr)oxides induced by phosphate at a molecular level.

## 1 Introduction

Reactions occurring on natural nanoparticles significantly influence numerous environmental processes, including the distribution of heavy metals in soil and water.^1^ Lead (Pb), a highly toxic heavy metal, poses substantial risks to both human health and environmental safety.^2^ Like other heavy metals, Pb interacts strongly with natural nanoparticles such as metal (hydr)oxides and natural organic matter (NOM), which govern the bioavailability and mobility of Pb in the environment.^1,3^

Great efforts have been dedicated to understanding and quantifying the surface reactions of heavy metals in soil and water. A key challenge lies in identifying the natural nanoparticles that most significantly contribute to metal binding, followed by the development of surface complexation models (SCMs) that are capable of describing and predicting metal speciation and distribution in the environment.^4−7^ This approach has been considerably successful, and models such as the multisurface model (MSM) can accurately predict the solid-solution distribution of heavy metals like copper (Cu) and cadmium (Cd).^7,8^ However, Pb presents a unique challenge, with reported outcomes often contradictory, and modeling attempts frequently unsuccessful. For instance, MSM tends to overestimate the concentration of soluble Pb in natural soils.^7,8^ The predominant role of NOM and metal (hydr)oxides in Pb adsorption also remains a topic of ongoing debate.^9−12^

Phosphate (PO_4_), which is commonly found in fertilizers and amendments, is renowned for its ability to immobilize cationic heavy metals such as Pb.^13,14^ Overlooking or underestimating the impact of PO_4_ on Pb immobilization could potentially explain the failure of MSM and similar models in accurately predicting Pb distribution.^15−17^ A variety of mechanisms have been proposed for PO_4_-mediated Pb immobilization on oxides, including synergistic electrostatic effect, formation of oxide-Pb-PO_4_ ternary surface complexes, and Pb-PO_4_ (surface) precipitation.^18−20^ However, the dominant mechanisms of PO_4_-mediated Pb immobilization on oxides remain unclear.^21,22^ Studies using X-ray absorption fine structure (XAFS) have produced conflicting results, with some suggesting that phosphate enhances Pb adsorption to oxides, while others propose that it promotes precipitation.^10,16,21,23−25^ Several studies claimed that PO_4_ forms ternary surface complexes with Pb on oxides.^16,26^ However, others argue that it is not necessary to consider ternary complexation when modeling PO_4_-enhanced Pb immobilization on goethite.^27^ Although various ternary structures have been proposed (Pb-bridged, P-bridged, and monodentate-O-sharing structure),^20^ the exact structure of the ternary complex of Pb-PO_4_ on iron (hydr)oxides is unclear.^16^ For goethite, a common soil iron (hydr)oxide, the existence, structure, and affinity of ternary Pb-PO_4_ complexes require further investigation.^27^ In addition, it is also challenging to distinguish and quantify Pb–PO_4_ precipitates that coexist with adsorbed Pb. In the presence of an oxide surface, surface precipitation due to heterogeneous nucleation or formation of a solid solution may occur, which may deviate from bulk solution precipitation.^28^ The lack of mechanistic understanding impedes the development of SCMs that can accurately predict Pb immobilization under the influence of PO_4_.

The Charge Distribution MUlti-SIte Complexation (CD-MUSIC) model is an advanced surface complexation model developed for ion adsorption to metal (hydr)oxides.^29^ In this study, batch adsorption experiments, CD-MUSIC modeling, XAFS analysis, and density functional theory (DFT) calculations were used to understand and quantify the contribution of electrostatic synergy, ternary complexes, and precipitation mechanisms to PO_4_-enhanced Pb immobilization on goethite under varying conditions. This molecular-level understanding and modeling approach developed would be helpful in resolving the controversy regarding Pb distribution in environmental samples and advance our ability to predict and manage Pb immobilization in the environment.

## 2 Materials and Methods

### 2.1 Chemicals

Reagents with a metal basis purity (≥99.99%, Aladdin Biochemical Technology, Shanghai, China) were used to prepare stock solutions of Pb(NO_3_)_2_, NaH_2_PO_4_, and NaNO_3_ with ultrapure water (>18.2 MΩ cm resistivity, Milli-Q IQ 7000, Millipore-Sigma, Billerica, MA, USA). Stocks were stored at 4 °C and diluted to target concentrations immediately before experiments.

### 2.2 Materials

Goethite and hydropyromorphite (HPM, Pb_5_(PO_4_)_3_OH) were synthesized following established methods.^30,31^ The synthesized goethite and HPM were confirmed by X-ray diffraction (XRD) and scanning electron microscopy (SEM) (Figure S1). The specific surface area of the goethite is 80.9 m^2^/g as measured by Brunauer–Emmett–Teller (BET) N_2_ adsorption. The point of zero charge (PZC, measured to be 9.2) and absolute charge curves of goethite were determined by acid–base titrations at variable ionic strengths paired with pH-static titration.^32^ More details are provided in S1 of the Supporting Information (SI).

### 2.3 Batch Adsorption Experiments

Separate and simultaneous adsorption of Pb (10–300 μM) and PO_4_ (200–400 μM) on goethite (1.3 g/L) was examined through batch experiments at equilibrium pH 3–7 in 10 mM NaNO_3_. Before use, the goethite suspension was purged with N_2_ overnight to eliminate bicarbonate. To mitigate the risk of excessive Pb-PO_4_ precipitation and aggregation,^33^ which could extend the time required to reach adsorption-precipitation equilibrium,^28^ PO_4_ was introduced first and Pb was added after 10 min. This strategy effectively reduces PO_4_ concentration in solution at the initial 10 min, as reported in a previous kinetics study.^34^ In another previous study, no discernible difference was found between simultaneous and sequential addition of PO_4_ and Cd to goethite with a 15 min interval.^35^ Finally, the total volume of each sample was adjusted to 20 mL using ultrapure water.

The samples were then subjected to pH adjustment using HNO_3_ and NaOH under N_2_ purging. The samples in sealed tubes were shaken at 180 rpm and 25 °C for a duration of 7 days. The pH was readjusted at 24 and 48 h to the desired pH values. At the end of the batch experiment (total equilibration time of 7 days), the equilibrium pH was recorded, and the samples were centrifuged at 15,000 *g* for 30 min at 25 °C, followed by filtration through 0.22 μm filters. The filtrates were acidified with HNO_3_ before further analysis. The concentrations of Pb and P were quantified using either inductively coupled plasma optical emission spectrometry (ICP-OES; 710, Agilent Technologies, USA) or inductively coupled plasma mass spectrometry (ICP-MS; ICAP-Q, Thermo Fisher Scientific, USA), depending on the concentrations.

### 2.4 CD-MUSIC Modeling

The CD-MUSIC model was employed to simulate Pb adsorption onto goethite, both without and with PO_4_.^31,36^ The Extended Stern model was used to depict the electrostatic structure of the goethite surface.^31^ Parameters such as site density, ion pair affinity constants, and Stern layer capacitances (*C*_1_ and *C*_2_) were sourced from the literature,^32^ with proton affinity constants set to match PZC.^29^ The model effectively simulated the surface charging behavior of the goethite used in this study (Figure S2). It was assumed that inner-sphere complexation of both Pb and PO_4_ occurs solely with singly coordinated surface sites.^31^ Both a monodentate and a bidentate inner-sphere surface complex of PO_4_ were considered.^37^ Their charge distribution values were adopted from Rahnemaie et al.,^37^ and their affinity constants were optimized.

In modeling Pb adsorption, 85% of singly coordinated sites were considered low-affinity, with the remaining 15% being high-affinity.^38,39^ Based on the literature and the stoichiometry derived from the extended X-ray absorption fine structure (EXAFS) analysis of this study, a bidentate Pb surface species and its hydrolysis species were considered for PO_4_-free systems.^39,40^ Their charge distribution values were adopted from Weng et al.,^7^ and their affinity constants were optimized. For systems with coexisting Pb and PO_4_, two scenarios were considered. In the first, an additional goethite-Pb-PO_4_ ternary complex was included (Model A), while in the second, this complex was omitted (Model B). The stoichiometry of the ternary complex was based on the EXAFS analysis, whereas the charge distribution and affinity constants were optimized based on experimental data of samples that did not contain HPM precipitates according to X-ray absorption near edge structure coupled with linear combination fitting (XANES-LCF). The exact structure of the Pb-PO_4_ ternary complex was further identified as the monodentate-O-sharing structure based on DFT calculations, and the charge distribution parameters optimized in CD-MUSIC modeling were validated with electrostatic potential (ESP) profiles and bond valence concept (BVC) obtained from cluster DFT calculations.

In modeling the formation of the Pb-PO_4_ precipitate, HPM was considered the most preferentially formed precipitate as a result of low solubility (Table S1). XANES results in our study also confirmed this (see Section 3.1). The solubility product (log *K*_sp_) of HPM was optimized based on the experimental data using samples containing HPM as identified with the XANES-LCF analysis. Interactions of the precipitate with charged minerals (goethite) may have changed its solubility compared with HPM in bulk solution.

Other thermodynamic constants used in this study are listed in Table S1. Differences between modeling and experimental values were described using the root mean square error (RMSE, Table S2). Model calculations and parameter optimizations were performed using ECOSAT 4.9 and FIT code.^41^ More details of CD-MUSIC modeling are provided in S2 of SI.

### 2.5 XAFS Characterizations of Pb and PO_4_ Adsorption on Goethite

XAFS analysis was employed to detect Pb and PO_4_ surface species on goethite. Selected samples from adsorption experiments (50–300 μM Pb and 200–400 μM PO_4_ at pH 5 or 7 in 10 mM NaNO_3_) were subjected to Pb L3-edge and P K-edge spectra collection. Pb L3-edge (*E*_0_ = 13035 eV) XAFS spectra were obtained with a 14W beamline at the Shanghai Synchrotron Radiation Facility (SSRF). P K-edge (*E*_0_ = 2145.5 eV) XANES spectra were collected with the 4B7A beamline of Beijing Synchrotron Radiation Facility (BSRF).

The Athena software was used for data preprocessing and LCF analysis.^42,43^ LCF analysis was performed on Pb L3-edge (−25 to 75 eV relative to *E*_0_) and P K-edge (−10 to 40 eV) XANES data with total weight constrained to 1 and no energy shift.^44,45^ The *k*^2^-weighted EXAFS data of Pb L3-edge analysis were fitted by FEFF using Artemis.^42,43^ The *k* and *R* range for all samples was 2–10 Å^–1^ and 1.2–4.0 Å, respectively. The wavelet transform of Pb EXAFS data was analyzed using wtEXAFS code.^46^ More details regarding the XAFS experiment and data processing are provided in S3 of SI.

### 2.6 DFT Calculations

Cluster and periodic DFT calculations were both employed for calculating Pb and PO_4_ adsorption on goethite (110) face based on chemical stoichiometry and surface species obtained with EXAFS analysis or/and CD-MUSIC modeling.^47−50^ For the cluster DFT calculations, an iron-dimer model was constructed, which has been validated by numerous studies for its effectiveness in simulating local binding structures.^37,51−55^ The cluster DFT calculations were executed using the Gaussian 16 software.^56^ Periodic DFT calculations were performed using the Vienna ab initio simulation package (VASP) 5.4.^57^ The goethite (110) surface slab model was constructed. The optimized cluster and periodic goethite surface model are depicted in Figure S3. The simultaneous use of cluster and periodic DFT enhances our understanding of the overall energy information while providing insight into the local binding properties.^58−60^

The existing literature suggests the potential existence of three distinct types of Pb-PO_4_ ternary complexes on metal (hydr)oxides: bidentate phosphorus-bridged, bidentate lead-bridged, and monodentate oxygen-sharing structures.^16,20^ The structures of ternary and bidentate Pb as well as bidentate and monodentate PO_4_ surface species were individually calculated. Bond length and wave function which obtained from cluster DFT were further subjected to BVC and ESP analysis, respectively.^32,47,61^ The adsorption energies (*E*_ads_) of ions onto goethite were computed based on periodic DFT. It is noteworthy that while the *E*_ads_ obtained from the periodic model are not strictly rigorous,^62^ they offer a valuable balance between computational efficiency and accuracy.^63,64^ More comprehensive details of the cluster and periodic DFT calculations can be found in S4 of SI.

## 3 Results and Discussion

### 3.1 Pb and PO_4_ (Co)Immobilization on Goethite

**Figure 1:**
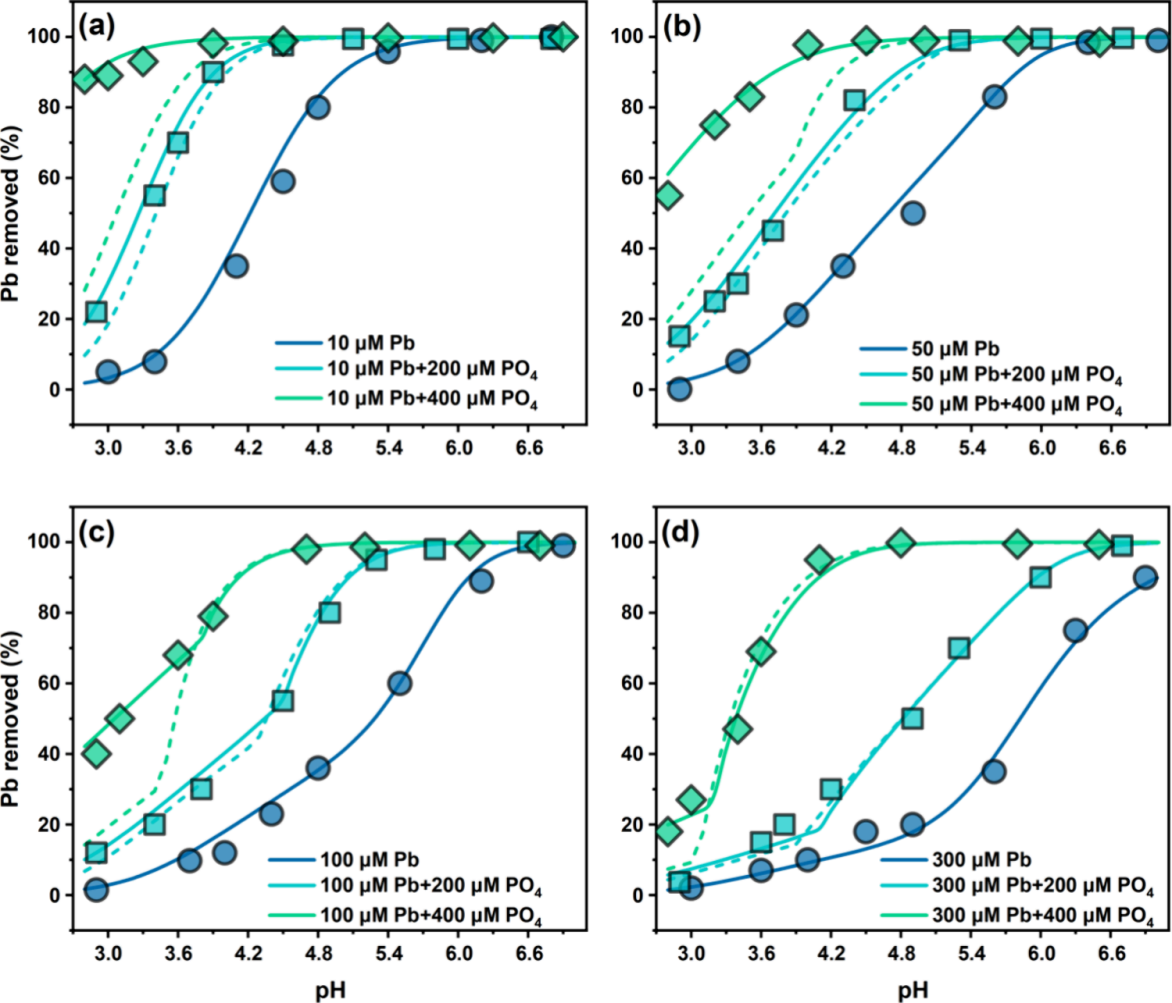
Adsorption envelopes of Pb on goethite in the absence and presence of PO_4_ at equilibrium pH 3–7. Data points represent the experimental results. Solid and dashed lines represent predictions of the CD-MUSIC model incorporating (Model A) and omitting (Model B) ternary surface complexation, respectively. Goethite: 1.3 g/L (105 m^2^/L). (a), (b), (c), and (d) are results for, respectively, initial Pb concentrations of 10, 50, 100, and 300 μM in the absence or presence of 200 or 400 μM PO_4_ in 10 mM NaNO_3_ background. CD-MUSIC parameters used are listed in Table 1. Root mean square error (RMSE) between experimental and modeling results of different systems is summarized in Table S2 of SI.

The adsorption envelopes of Pb on goethite at varied pH values in the absence and presence of PO_4_ are depicted in Figure 1, and the corresponding adsorption envelopes of PO_4_ can be found in Figure S4. Consistent with previous research, Pb adsorption on goethite increased strongly with the increase in pH (Figure 1).^39,40,65−67^ The plateau of the adsorption envelope, indicating near-complete adsorption, appears at pH 5.5 for an initial Pb concentration of 10 μΜ and shifts to a higher pH (e.g., pH 6.3 at 100 μΜ Pb) as the initial Pb concentration increases. With the addition of PO_4_, Pb adsorption significantly increases in a PO_4_ concentration-dependent manner (Figure 1). Near-complete removal shifts to lower pH values with PO_4_ addition (e.g., plateau shifts from pH 6.3 to pH 5.4 at 200 μΜ PO_4_ addition with 100 μΜ Pb), and the shift increases as the initial PO_4_ level increases and the initial Pb level decreases.

Regarding PO_4,_ at pH 3, approximately 100, 80, and 60% of added PO_4_ was adsorbed with initial PO_4_ concentrations of 200, 300, and 400 μM respectively without Pb, and PO_4_ adsorption decreases as pH increases (Figure S4). The presence of Pb enhances PO_4_ adsorption, with the effect increasing as the Pb concentration increases.

### 3.2 Geochemical Modeling

#### 3.2.1 Bidentate Pb Complexation

The CD-MUSIC model was employed to simulate the adsorption envelopes of Pb on goethite, with the aim of elucidating the underlying mechanisms of Pb adsorption on goethite in the presence of PO_4_. In PO_4_-free systems, our calculations incorporated bidentate Pb adsorption (≡(FeOH)_2_Pb^+^) according to previous and current EXAFS results (see Section 3.3.1), and its hydrolysis complex was also considered.^39,40,68,69^ To account for the heterogeneous distribution of functional groups on goethite, we assumed that 85% of singly coordinated sites on goethite were low-affinity and 15% were high-affinity, representing the differences between (110) and (021) faces in the model.^38,39,70−72^ The parameters used can be found in Table 1.

**Table 1:** Surface Complexation Reactions and Corresponding Constants Applied in the CD-MUSIC Model Describing Pb and PO_4_ Co-Adsorption on Goethite

surface complexation reactions	CD-values			log *K*
	Δ*z*_0_	Δ*z*_1_	Δ*z*_2_	
*proton binding reactions*				
FeOH^–0.5^ + H^+^ ↔ FeOH_2_^+0.5^	1	0	0	9.20^a^
Fe_3_O^–0.5^ + H^+^ ↔ Fe_3_OH^+0.5^	1	0	0	9.20^a^
*ion pairs*				
FeOH^–0.5^ + Na^+^ ↔ FeOHNa^+0.5^	0	1	0	–0.60^a^
Fe_3_O^–0.5^ + Na^+^ ↔ Fe_3_ONa^+0.5^	0	1	0	–0.60^a^
FeOH^–0.5^ + H^+^ + NO_3_^–^ ↔ FeOH_2_NO_3_^–0.5^	1	–1	0	8.62^a^
Fe_3_O^–0.5^ + H^+^ + NO_3_^–^ ↔ Fe_3_OHNO_3_^–0.5^	1	–1	0	8.62^a^
*inner-sphere complexation*				
2FeOH^–0.5^ + PO_4_^3–^ + 2H^+^ ↔ Fe_2_O_2_PO_2_^–2^ + 2H_2_O	0.46	–1.46	0	29.31^b^
FeOH^–0.5^ + PO_4_^3–^ + 2H^+^ ↔ FeOPO_2_OH^–1.5^ + H_2_O	0.28	–1.28	0	27.35^b^
2FeOH_L_^–0.5^ + Pb^2+^ ↔ (FeOH_L_)_2_Pb^+^	1.15	0.85	0	9.64^b^
2FeOH_H_^–0.5^ + Pb^2+^ ↔ (FeOH_H_)_2_Pb^+^	1.15	0.85	0	12.45^b^
2FeOH_L_^–0.5^ + Pb^2+^ + H_2_O ↔ (FeOH_L_)_2_PbOH^0^ + H^+^	1.15	–0.15	0	2.10^b^
2FeOH_H_^–0.5^ + Pb^2+^ + H_2_O ↔ (FeOH_H_)_2_PbOH^0^ + H^+^	1.15	–0.15	0	3.62^b^
2FeOH_L_^–0.5^ + Pb^2+^ + PO_4_^3–^ + 2H^+^ ↔ (FeO_L_)_2_HPbPO_3_H^0^ + H_2_O	0.60	0.40	0	33.31^c^
2FeOH_H_^–0.5^ + Pb^2+^ + PO_4_^3–^ + 2H^+^ ↔ (FeO_H_)_2_HPbPO_3_H^0^ + H_2_O	0.60	0.40	0	35.05^c^

a Adopted from Hiemstra and Van Riemsdijk.^32^

b CD values were adopted from Weng et al.^7^ and Rahnemaie et al.,^37^ while log *K* values were optimized based on experimental data in this study.

c CD and log *K* values were optimized based on the experimental data in this study; site density of ≡FeOH^–0.5^ and ≡Fe_3_O^–0.5^ is 3.45 and 2.7 sites/nm^2^, respectively, which was derived according to the lattice analysis of goethite performed by Hiemstra et al.^36^ Low (≡FeOH_L_^–0.5^) and high (≡FeOH_H_^–0.5^) affinity sites account for 85 and 15% of the total FeOH^–0.5^ sites, respectively. Capacitance of the first and second Stern layer (*C*_1_ and *C*_2_) is 0.85 and 0.75 F/m^2^, respectively. The stoichiometric number of the Pb-PO_4_ ternary complex is based on the EXAFS analysis of this study.^16^ More details of the CD-MUSIC approach and the parameter optimization are provided in S2 of SI.

The model demonstrated a satisfactory fit for the experimental Pb adsorption data on goethite in the absence of PO_4_ (Figure 1 and Table S2). Furthermore, using the same parameters, the CD-MUSIC model accurately described two recently published Pb-goethite data sets (Figure S5).^39,71^ This suggests that the parameters derived in this study are able to predict Pb adsorption over a wide range of conditions and on goethite materials prepared in different laboratories.

#### 3.2.2 Ternary Pb-PO_4_ Complexation

In Pb-PO_4_ coexisting systems, Model B that did not include Pb-PO_4_ ternary complexes underestimated Pb adsorption when 400 μM PO_4_ was added (RMSE = 20.0%, Table S2), although it was successful in predicting Pb adsorption when 200 μM PO_4_ was added (Figure 1). On the other hand, Model A that incorporated ternary Pb-PO_4_ complexes can accurately describe Pb adsorption in all systems (RMSE = 2.2–2.7%), indicating that the inclusion of ternary surface species is crucial for improving the accuracy of Pb adsorption predicted, particularly at high PO_4_ levels.^16,17,50^ The optimized model parameters for this ternary complex are listed in Table 1.

Contrary to our findings, Xie and Giammar^27^ concluded that ternary complexes were not necessary for simulating PO_4_-enhanced Pb adsorption on goethite-coated sand. To reconcile this, we simulated their data by using our model. While minor adjustments of the log *K* values of bidentate and ternary Pb complexes were made (Table S3), other parameters were kept consistent with those used in modeling our own data. Our model accurately replicated their experimental data. Ternary complexes are negligible in most samples (0–6%) due to low initial PO_4_ levels (0.0008–12 μΜ, Table S4) in their data sets. Only one sample with a high PO_4_ level (1200 μM) exhibited a significant presence of ternary complexed Pb (∼17%), while precipitation was also a key factor in this sample (∼20%). Consequently, the contrasting conclusion between the current study and Xie and Giammar^27^ could be attributed to the difference in experimental conditions, in this case, PO_4_ concentration.

Utilizing the CD-MUSIC model parameters for Pb adsorption on goethite established in this study and model parameters for Cd adsorption on goethite reported in the literature,^5^ we compared the tendency of ternary complex formation in the presence of PO_4_ between these two metals (Figure S6). The results confirmed that the formation of these ternary complexes on goethite is of much greater significance for Pb as compared to Cd.^5^ In addition, calculations using the literature model parameters for Pb, Cd, Cu, and Zn adsorption on ferrihydrite also demonstrated a higher likelihood for Pb and PO_4_ of forming ternary complexes as compared to Cd, Zn, and Cu.^16,17,50^ The results suggest that omitting Pb-PO_4_ ternary complex formation may significantly underestimate Pb immobilization in environmental samples, especially at a high PO_4_ loading.

#### 3.2.3 Precipitation

To improve the modeling when Pb-PO_4_ precipitate is present, the solubility product (log *K*_sp_) of HPM was optimized, resulting in a value of −82.02, which is 1.25–5.23 orders of magnitude lower than the values (−76.79 to −80.77) reported in the literature.^30,33,73,74^ This lower log *K*_sp_ allowed for accurate modeling of Pb removal in all treatments of the batch experiment (Figure 1). For the treatment of 300 μM Pb and 400 μM PO_4_, in which the precipitation is the most significant among the samples, the RMSE between the model predictions and the experimental data is 2.3% (Figure S7). In comparison, using the literature log *K*_sp_ values resulted in higher RMSEs of 8.6–21.7%.

The lower value of log *K*_sp_ optimized compared to that of HPM in bulk solution may be attributed to the enhanced precipitation by goethite.^18,20,40,75^ As shown by Shi et al.,^19^ Pb-PO_4_ precipitates in the presence of goethite do not involve surface sites and they suggested that the precipitate was adsorbed to the charged mineral as a result of electrostatic interaction. Because our XAFS results also showed no direct involvement of surface sites in Pb-PO_4_ precipitate, and the structure of this precipitate is similar to that of HPM, we treated this precipitation reaction in a similar way to that in the bulk solution in the CD-MUSIC modeling, while the solubility product (log *K*_sp_) was adjusted. Similarly, Komárek et al.^40^ improved their modeling accuracy of Pb immobilization on charged minerals (hematite and lepidocrocite) by lowering the log *K*_sp_ value of Pb carbonate minerals. Heterogeneous nucleation may have promoted the formation of Pb-PO_4_ precipitation in the presence of goethite instead of forming a solid solution. It is important to note that for simplicity, we employed a method to elucidate the effect of the presence of goethite on Pb-PO_4_ precipitation without further distinguishing between precipitates closely associated with the oxide surface or in the solution.

### 3.3 Contribution of Different Mechanisms to PO_4_-Induced Pb Immobilized on Goethite

#### 3.3.1 Analysis Based on Pb L3-Edge XANES

**Figure 2:**
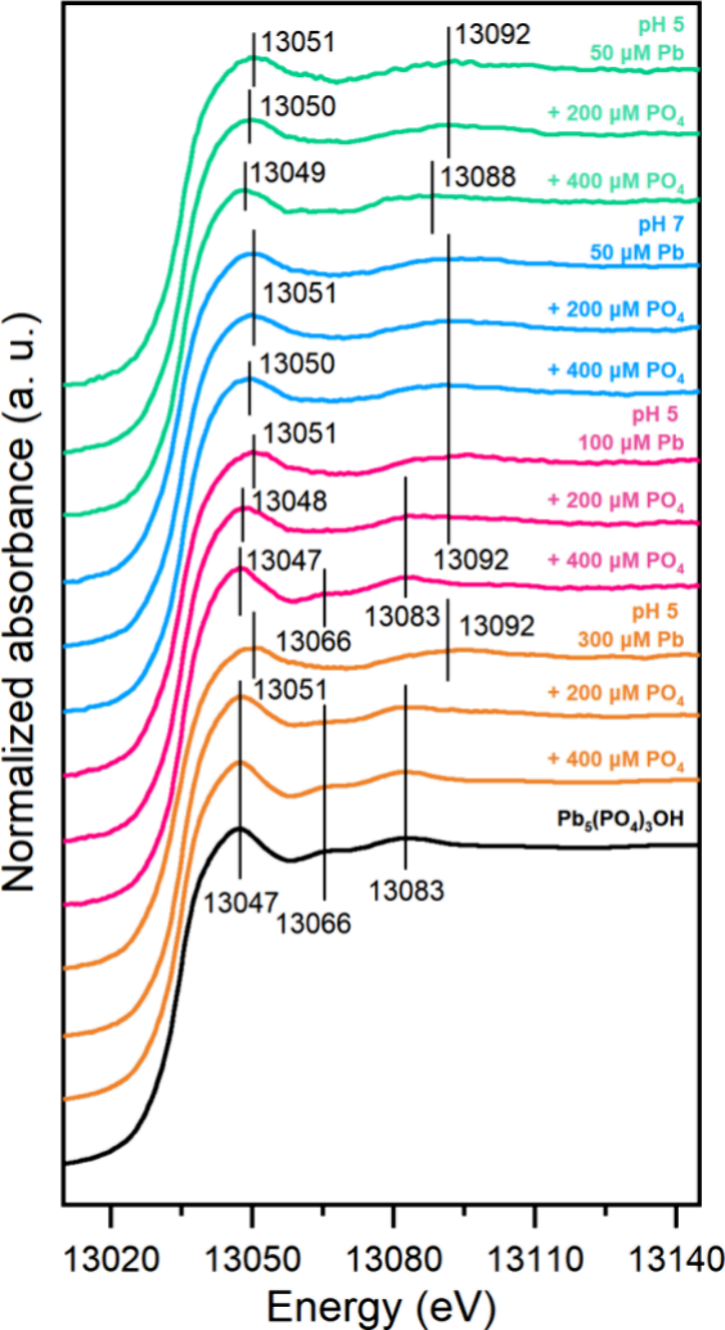
Normalized XANES spectra of Pb immobilized on goethite under initial Pb and PO_4_ concentration of 50–300 and 200–400 μM, respectively, at pH 5 or 7 in 10 mM NaNO_3_. Goethite: 1.3 g/L (105 m^2^/L). Different colored lines represent samples with different Pb initial concentrations or equilibrium pH, and the black line represents hydropyromorphite (HPM). Peaks at 13051, 13092 eV and 13047, 13066, 13083 eV represent characteristics of bidentate adsorption and precipitation, respectively, while other peaks represent an energy shift which could be attributed to the formation of Pb-PO_4_ ternary complex.

The coordination environment and speciation of Pb on goethite in the absence and presence of PO_4_ were investigated by using XAFS. Figure 2 displays the Pb L3-edge XANES spectra of samples with 50–300 μM initial Pb and 200–400 μM initial PO_4_ concentrations at pH 5 and pH 7, along with the spectra of synthesized HPM. All samples without PO_4_ showed peaks at 13051 and 13092 eV, corresponding to bidentate adsorbed Pb, consistent with previous studies.^19,26^ HPM exhibited distinct peaks at 13047, 13066, and 13083 eV. In addition to these well-defined peaks associated with bidentate adsorbed or precipitate-Pb, some samples exhibited different peaks. For instance, upon the addition of 200 and 400 μM PO_4_, the peak at 13051 eV observed for Pb without PO_4_ shifted to 13050 and 13049 eV, respectively. Furthermore, under treatment with 400 μM PO_4_, the peak at 13092 eV for Pb in the absence of PO_4_ shifted to 13088 eV. These shifts may be attributed to the formation of Pb-PO_4_ ternary complexes.^19^

LCF analysis was performed to quantify Pb speciation on goethite. Following Shi et al.,^19^ samples without PO_4_ and the synthesized HPM sample served as references for bidentate Pb surface species and Pb-PO_4_ precipitate, respectively. Using these two end members, the LCF of the Pb L3-edge spectra showed good fitting quality (Figure S8a, Table 2). However, it is noteworthy that around the edge crest position (13,049–13,063 eV), the fitting quality for the sample with 50 μM Pb and 400 μM PO_4_ at pH 5 was mediocre. The forced inclusion of HPM in the fitting did not enhance the fitting quality (Figure S9). This could be attributed to the significant distinct coordination environment compared to the bidentate adsorption or precipitate-Pb of this sample, possibly due to the significant presence of ternary complexes in this sample.^19,26^

**Table 2:** Quantitative Analysis of Pb Species on Goethite in the Presence of PO_4_ Using Pb L3-Edge XANES-LCF and CD-MUSIC Modeling^g^

sample			LCF excluded ternary complex^a^				LCF included ternary complex^b^					CD-MUSIC (%)				
pH	Pb (μM)	PO_4_ (μM)	species (%)		*R*-factor^e^	χ^2^^f^	species (%)			*R*-factor	χ^2^	Model A^cd^			Model B^cd^	
			ads.	pre.			ads.	pre.	ter.			ads.	pre.	ter.	ads.	pre.
5	50	200	100(2.6)	0	0.0008	0.0205	100(2.4)	0	21.2(6.4)	0.0006	0.0165	100	0	20.6	96.8	3.2
		400	100(3.7)	0	0.0016	0.0393						100	0	68.0	74.3	25.7
7	50	200	100(1.9)	0	0.0004	0.0102	100(1.8)	0	9.9(4.7)	0.0003	0.0093	100	0	3.9	98.3	1.7
		400	90.4(2.7)	9.6	0.0008	0.0211	89.2(8.0)	10.8	21.6(5.3)	0.0006	0.0173	89.9	10.1	10.1	84.1	15.9
5	100	200	78.8(2.6)	21.2	0.0007	0.0197	77.4(5.6)	22.6	26.8(3.7)	0.0005	0.0138	61.2	38.8	6.9	55.7	44.3
		400	60.6(4.2)	39.4	0.0021	0.0532	61.7(10.2)	38.3	42.1(6.7)	0.0011	0.0282	60.7	39.3	41.7	37.5	62.5
	300	200	33.9(3.0)	66.1	0.0010	0.0422	33.4(9.8)	66.6	9.9(6.5)	0.0009	0.0255	38.1	61.9	0.5	37.6	62.4
		400	14.9(1.5)	85.1	0.0004	0.0061	18.0(3.9)	82.0	12.3(2.5)	0.0001	0.0039	18.8	81.2	8.9	13.6	86.4

a The LCF analysis employs two references: A sample with Pb solely adsorbed on goethite and HPM, distinguishing only between adsorbed and precipitated Pb.

b The LCF analysis incorporates three references: A sample with Pb solely adsorbed on goethite, HPM, and a sample with 50 μM Pb and 400 μM PO_4_ at pH 5. Postfitting, 68% of the sample with 50 μM Pb and 400 μM PO_4_ at pH 5 was treated as the ternary complex content, with the remaining 32% as bidentate adsorption. This fitting differentiates between bidentate adsorption, precipitation, and ternary complexation of Pb;

cd CD-MUSIC modeling is performed with and without considering Pb-PO_4_ ternary complexes, respectively.

e The goodness of fit parameter is defined as *R*-factor = ∑_*i*_(exp. – fit)^2^/∑_*i*_(exp.)^2^, where “exp.” represents the experimental value of XANES, and “fit” signifies LCF results.

f χ^2^ = ∑_i_[(exp. – fit)^2^/fit]. Ads.: total adsorbed. Ter.: ternary complexed. Pre.: precipitated. The total Ads. in Model A comprise the sum of bidentate and ternary complexation of Pb on goethite. Uncertainties of precipitate are equal to those of total adsorbed. The LCF-fitted XANES spectra are shown in Figure S8. The correlations and RMSEs between the LCF and CD-MUSIC model are presented in Table S5.

g The numbers in parentheses represent the uncertainties of the fitting value.

According to the CD-MUSIC modeling considering ternary complexation (Model A), the sample mentioned above (50 μM Pb and 400 μM PO_4_ at pH 5) demonstrates a high contribution of Pb-PO_4_ ternary complexes (68%, Table 2). Consequently, we endeavored to quantify these ternary complexes via XANES-LCF for all of the samples, incorporating the aforementioned sample as a reference for Pb-PO_4_ ternary complexes after subtracting the contribution of the other 32% as bidentate adsorbed Pb. The results of this fitting process using three end members (bidentate Pb adsorption, ternary Pb-PO_4_ adsorption, and precipitation), are presented in Figure S8b and Table 2. The fitting quality (*R*-factor) improved for all samples compared with the fitting considering only bidentate-adsorbed and precipitate Pb. Including ternary complexes did not change the overall adsorption and precipitation contribution but shifted the adsorbed amount from bidentate to ternary-complex adsorbed Pb. The unaffected precipitated amount when including the ternary complex suggests that the spectral characteristics of the sample (50 μΜ Pb and 400 μΜ PO_4_ at pH 5) are more inclined toward ternary adsorption, not precipitation. The contribution of Pb-PO_4_ ternary complexes fitted aligns well with those predicted by the CD-MUSIC model (*R*^2^ = 0.757; RMSE = 9.1%, Table S5).

The LCF results using both two and three end members revealed the absence or small amount (<∼10%) of HPM precipitation when the initial Pb concentration was 50 μM, even at high pH (7) and high PO_4_ concentration (400 μM) (Table 2). Similarly, Tiberg et al.^16^ also found no evidence of precipitation at 28 μM Pb and 600 μM PO_4_ in the presence of ferrihydrite. However, with 100–300 μM initial Pb, significant Pb-PO_4_ precipitation occurred in all PO_4_-containing samples analyzed (∼20–40% at 100 μM Pb; ∼65–85% at 300 μM Pb). According to the LCF using three end members, the formation of Pb ternary complexes tends to occur at low pH and high PO_4_ concentrations. For example, in samples of 50 μM Pb and 200 μM PO_4_, the ternary Pb concentration was significantly higher at pH 5 (21.2%) than at pH 7 (9.9%). Moreover, in the samples of 100 μM Pb at pH 5, the ternary Pb concentration was significantly higher at 400 μM PO_4_ (42.1%) than at 200 μM PO_4_ (26.8%).

#### 3.3.2 Analysis Based on P K-Edge XANES

The speciation of PO_4_ and its relation to Pb speciation on goethite were investigated by using P K-edge XANES spectroscopy for samples with 400 μM PO_4_ without or with 50–300 μM Pb at pH 5 (Figure S10). The spectral features of HPM and goethite-sorbed PO_4_ were distinct (Figure S10a). The white line peak of PO_4_ adsorbed on goethite appeared at 2154.2 eV, whereas the white line peak of HPM occurred at 2153.7 eV but with a lower intensity. In addition, a shoulder peak at 2160.7 eV, similar to chloropyromorphite was observed for HPM.^76^ This shoulder peak is invisible for 50 and 100 μM Pb treatment (400 μM PO_4_), but this peak is clear to see for the sample of 300 μM Pb and 400 μM PO_4_ treatment.

LCF analysis of P K-edge spectra used goethite with only PO_4_ and HPM as the references for adsorbed PO_4_ and PO_4_ in a Pb-PO_4_ precipitate, respectively. The results showed good fitting quality (Figure S10b and Table S6). Precipitated PO_4_ was absent at 50 μM Pb, and only 7.7% of PO_4_ was precipitated at 100 μM Pb, but it increased significantly to 39.6% at 300 μM Pb (Table S6). The P:Pb ratio of the precipitate is 0.588–0.619, obtained from the P K-edge and Pb L3-edge LCF analysis results, consistent with the 0.6 ratio based on the stoichiometry of HPM (Pb_5_(PO_4_)_3_OH), strongly indicating that HPM is the predominant mineral in the precipitation phase (Table S6). Based on the P:Pb ratio of HPM (0.6) and the LCF-derived precipitated PO_4_ percentages, the levels of precipitated Pb were estimated to be 0, 36.8, and 80.2% in samples with 50, 100, and 300 μM Pb under 400 μM PO_4_ addition at pH 5, respectively. These values agree well with the values of 0, 38.3, and 82.0% from the Pb L3-edge LCF results using three end members or 0, 39.4, and 85.1% using two end members.

#### 3.3.3 Quantification with the CD-MUSIC Model

Both Model A and Model B of the CD-MUSIC model, which consider and omit ternary complexation, respectively, were used to calculate the same XAFS-analyzed samples. Both models included HPM precipitation with log *K*_sp_ = −82.02. As shown in Table S5, for distinguishing between precipitation and total adsorption, Model A closely aligned with the LCF analysis results of Pb L3-edge XANES spectra (*R*^2^ = 0.957–0.958, RMSE = 6.0–6.5%), while Model B showed less agreement (*R*^2^ = 0.870–0.897, RMSE = 11.9–15.0%). Model A also better matched the LCF results of the P K-edge regarding PO_4_ speciation than Model B. These results underscore the importance of including ternary complexes in the model and affirm the reliability of the CD-MUSIC model. Omitting the ternary complexation in the modeling would lead to the overestimation of Pb-PO_4_ precipitation contribution to PO_4_-induced Pb immobilization, especially at relatively low Pb loadings (Table 2).

The CD-MUSIC model (Model A) was further applied to quantify the contribution of different mechanisms to PO_4_-induced Pb immobilization under the experimental conditions of all treatments in the batch experiment (Figure S11). The results showed that before Pb-PO_4_ precipitation took place, at all Pb concentrations, adding 200 μM PO_4_ enhanced mainly Pb bidentate complexation through electrostatic synergy, rather than forming Pb-PO_4_ ternary complexes. This explained the similarity in the results between Model A and Model B at a low phosphate level (200 μM PO_4_). For 400 μM PO_4_ with low Pb levels (10 and 50 μM), Pb removal was mainly through the formation of ternary complexes at lower pH (below 5.5). However, at higher pH (above 5.5), the contribution of ternary complexes decreased considerably. At high Pb levels (100 and 300 μM), as pH and initial Pb concentration increased, the contribution of adsorption (sum of bidentate and ternary complex) decreased while precipitation became the dominant PO_4_-induced Pb removal mechanism.

### 3.4 Structure, Energy, and Charge Distribution of Pb Surface Species

#### 3.4.1 Structure of Bidentate Complex

To elucidate the structure of Pb adsorbed on goethite, EXAFS shell-by-shell fitting was conducted on samples with 50 μΜ Pb and varying PO_4_ concentrations at pH 5 and 7, in which no or minimal HPM precipitation was detected by XANES-LCF and CD-MUSIC modeling. For PO_4_-free samples, the Pb–O coordination number (CN) of 1.8–2.0 for the first shell indicated bidentate complexation at pH 5 and 7 (Table 3). The second and third shell Fe are at 3.35–3.37 and 4.01–4.02 Å, respectively, conforming with bidentate mononuclear and binuclear complexes on the (021) and (110) face of goethite, aligning with previous EXAFS studies (Table S7).^68−70,77,78^ However, the CN of Pb–Fe on the (110) face is ambiguous, with our samples showing a CN of 0.4–0.8 (Table 3), deviating from the theoretical value of 2. This issue was also reported by other relevant EXAFS studies.^69,70,77,78^ One possible reason for this discrepancy is that EXAFS is relatively insensitive to atoms at greater distances, resulting in weaker signals from the third shell Fe atoms at distances larger than 3.9 Å.

**Table 3:** Coordination Environment Parameters Obtained from Pb L3-Edge Extended X-ray Absorption Fine Structure (EXAFS) Analysis for Pb Adsorption on Goethite in the Absence or Presence of PO_4_^g^

sample information	path	CN^a^	*R*^b^	σ^2^^c^	Δ*E*_0_^d^	*R*-factor^e^
			(Å)	(Å^2^)	(eV)	
pH 5 50 μM Pb	Pb–O	1.8(3)	2.28(1)	0.003(2)	–7.7	0.020
	Pb–Fe	0.8(2)	3.37(1)			
	Pb–Fe	0.4(3)	4.02(5)			
pH 5 50 μM Pb+200 μM PO_4_	Pb–O	1.9(3)	2.27(1)	0.007(2)	–9.9	0.042
	Pb–P	0.8(3)	3.25(3)			
	Pb–Fe	0.7(4)	3.91(2)			
pH 5 50 μM Pb+400 μM PO_4_	Pb–O	2.2(3)	2.31(1)	0.011(3)	–9.9	0.033
	Pb–P	0.7(4)	3.55(5)			
	Pb–Fe	0.2(1)	3.97(3)			
pH 7 50 μM Pb	Pb–O	2.0(1)	2.29(1)	0.005(1)	–5.6	0.003
	Pb–Fe	0.5(1)	3.35(1)			
	Pb–Fe	0.8(1)	4.01(1)			
pH 7 50 μM Pb+200 μM PO_4_	Pb–O	2.7(5)	2.32(1)	0.010(3)	–4.9	0.027
	Pb–Fe	0.3(1)	3.38(2)	0.001^f^		
	Pb–Fe	0.7(2)	3.99(2)	0.001^f^		
pH 7 50 μM Pb+400 μM PO_4_	Pb–O	2.0(2)	2.30(1)	0.006(2)	–6.8	0.011
	Pb–Fe	0.3(2)	3.32(3)			
	Pb–Fe	0.7(3)	3.98(3)			

a Coordination number.

b Interatomic distance.

c Debye–Waller factor.

d Energy shift threshold.

e Goodness-of-fit parameter: the quality of the fit was assessed using the *R*-factor, calculated as Σ(χ_data_ – χ_fit)_^2^/Σ(χ_data_)^2^, where χ_data_ and χ_fit_ represent the experimental and calculated structure factors, respectively. A value of the *R*-factor below 0.05 indicates a good fit quality;

f Constrained in fitting; The passive amplitude reduction factor (*S*_0_^2^) for all samples was set to 0.8. The experimental and best-fitted EXAFS spectra are provided in Figure S12 in SI. The details in the FEFF fitting of EXAFS are provided in S3 of SI.

g The numbers in parentheses represent the uncertainty of the last digit of the fitted value.

#### 3.4.2 Structure and Energy of the Ternary Complex

For PO_4_-containing samples at pH 7, using Fe as the second shell gave good EXAFS shell-by-shell fits and the results are similar to PO_4_-free samples (Table 3 and Figure S12), reflecting a low level of ternary Pb-PO_4_ complex in these samples and dominance of bidentate complexes, as also predicted by the CD-MUSIC model and analyzed by XANES. However, for PO_4_-containing samples at pH 5, using Fe as the second shell resulted in poor fits for FEFF fitting, with Pb–Fe distances differing from those in PO_4_-free samples (*R*-factor = 0.044–0.078, Table S8 and Figure S13). Enhanced backscattering at *R* + Δ*R* ≥ 3.5 Å was also observed in wavelet transform analysis (Figure S14). Coincidentally, the CD-MUSIC model also indicated high fractions of the ternary Pb-PO_4_ complex in these samples (Table 2). This evidence suggested that Pb-PO_4_ co-complexation altered the Pb second shell coordination environment compared to PO_4_-free samples and PO_4_-containing samples at pH 7.^16^ This alteration prevents the use of the same fitting approach for PO_4_-containing samples at pH 5 as that in other samples. Instead, using P as the second shell for FEFF fitting gave excellent fits for these samples (*R*-factor = 0.033–0.042, Table 3), providing strong evidence of the formation of a ternary complex between Pb and PO_4_ on goethite.^17,26^ The Pb–P CN of 0.7–0.8 indicated the presence of one P atom adjacent to Pb. The Pb–P distance is 3.55 Å in the typical ternary-complex-enriched sample (50 μM Pb and 400 μM PO_4_ at pH 5), in which the ternary complex accounted for 68.0% of Pb adsorbed (Table 2) as calculated by the CD-MUSIC model. Co-complexation of Pb and PO_4_ on other minerals such as TiO_2_ and ferrihydrite^26^ and goethite with other anions or organic ligands such as sulfate, carbonate, and humic acids^69,78,79^ have also been investigated in the previous literature.

**Figure 3:**
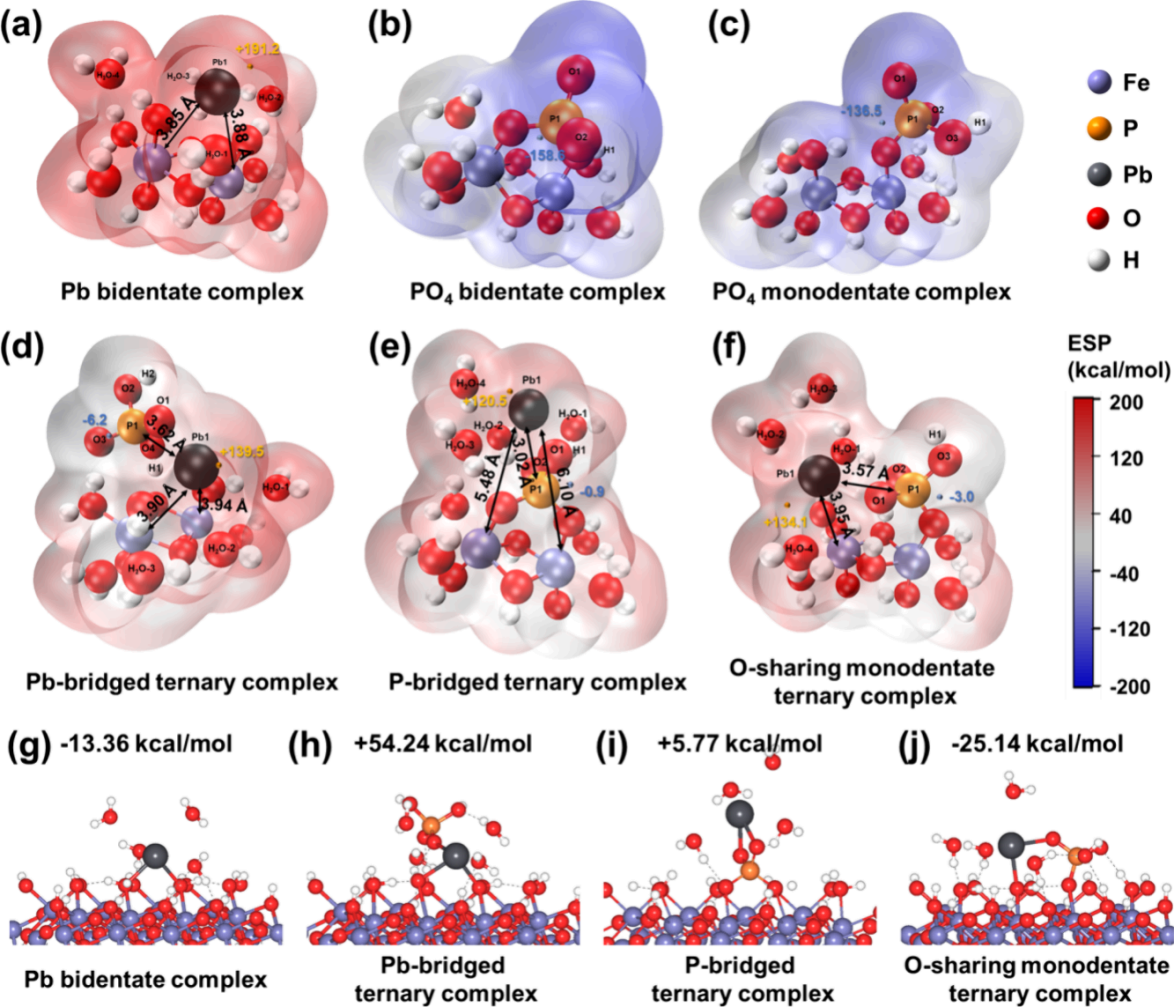
Cluster density functional theory (DFT)-optimized local binding structures and their electrostatic potential (ESP)-mapped molecular van der Waals surface of Pb and PO_4_ surface species adsorbed on iron clusters (a–f), as well as periodic DFT-calculated adsorption energy (*E*_ads_) on gothite (110) face (g–j). In parts a–f, the small yellow and blue dots represent the surface local minima and maxima of ESP related to Pb and P atoms, respectively. The blue area denotes the negative region of ESP, while the red area denotes the positive region of ESP. In parts g–j, the black character represents the adsorption energies. The bond lengths derived from cluster and periodic DFT are consistent; they are not indicated in the periodic DFT images. For details in DFT calculations and ESP analysis, please refer to S4 of SI.

To elucidate the binding structure and energies of the Pb-PO_4_ ternary complexes on goethite, cluster and periodic DFT calculations were performed. These calculations provided more detailed molecular information about their geometry and thermodynamics than EXAFS analysis alone. From an energetic perspective, the adsorption energies (*E*_ads_) calculated by periodic DFT of the various ternary complexes revealed that the monodentate-O-sharing ternary complex is thermodynamically the most stable, with an *E*_ads_ of −25.14 kcal/mol. In contrast, the Pb-bridged and P-bridged ternary complexes exhibited positive *E*_ads_ values of +54.24 and +5.77 kcal/mol respectively, indicating their relative instability (Figure 3g–j). Structurally (Figure 3d–f), the cluster DFT calculated monodentate-O-sharing and Pb-bridged ternary structures presented Pb–Fe and Pb–P distances of 3.95 and 3.57 and 3.92 and 3.62 Å, respectively. These results are in concordance with the EXAFS-derived distances of 3.97 and 3.55 Å for a sample containing 50 μM Pb and 400 μM PO_4_ at pH 5. However, the P-bridged ternary structure diverged, with cluster DFT-calculated Pb–Fe and Pb–P distances of 5.48 and 3.03 Å.

#### 3.4.3 Charge Distribution of Ternary Complex

To better identify the charge distribution of Pb surface species, cluster DFT results were analyzed by ESP (electrostatic potential) and BVC (bond valence concept) methods. Figure 3 shows the visualized ESP analysis with the ESP areas for P, Pb, and adjacent water molecules above goethite summarized in Table S9. The bidentate complex had the highest positive ESP near Pb (+191.2 kcal/mol, Figure 3a), while the ternary complexes with PO_4_ had lower positive ESP maxima for Pb (+139.5, +120.5, and +134.1 kcal/mol for Pb-bridged, P-bridged, and monodentate-O-sharing complexes, respectively, Figure 3d–f). This indicates that ternary complexes would form more easily on the net positively charged (when pH below PZC) goethite surface than bidentate complexes.

The CD-MUSIC model reveals that the Δ*z*_0_ of the bidentate Pb surface complex is 1.15 valence units (v.u.), while that of the Pb-PO_4_ ternary complex is 0.60 v.u. (Table 1). This suggests that the ternary complexation of Pb with PO_4_ reduces the contribution of the positive charge to the 0-plane. Among the three complexes computed by the BVC method, a decrease in charge distributed to the 0-plane was solely observed in the P-bridged and monodentate-O-sharing ternary structures, not in the Pb-bridged structure (Table S9). In the monodentate-O-sharing ternary structure, the complexation of PO_4_ can reduce the Δ*z*_0_ charge of one adsorbed Pb species from 1.28 v.u. (Pb-bidentate complex in BVC) to 0.64 v.u.

As indicated, DFT and EXAFS analyzed structure parameters have eliminated the possibility of phosphorus-bridged ternary complexes, while BVC analysis (based on cluster DFT) and the CD-MUSIC model analyzed that the charge profiles have dismissed lead-bridged complex formations. Furthermore, the *E*_ads_ calculated via periodic DFT strongly suggest that the monodentate-O-sharing ternary complex is the most energetically favorable among the various ternary complex structures. This evidence compellingly supports the conclusion that the monodentate-O-sharing structure is the predominant species of the Pb-PO_4_ ternary complex on goethite surfaces. This finding resolves the uncertainties previously noted by Tiberg et al.,^16^ who were unable to conclusively exclude the formation of either lead-bridged or monodentate-O-sharing ternary complexes on ferrihydrite based solely on EXAFS data. Our findings demonstrate that the former structure is inconsistent with both the charge profile and thermodynamic considerations through DFT calculations and CD-MUSIC modeling.

The charge distributed to one-plane (Δ*z*_1_) for Pb and PO_4_ surface species in CD-MUSIC modeling (Table 1) was compared with those derived with BVC analysis based on cluster DFT calculations, as well as with the difference between the positive and negative potential area from ESP analysis (APEP-ANEP) for these surface species, revealing high correlations (*R*^2^ = 0.95–0.97, Figure S15). The correlation with DFT calculations further validates the charge distribution parameters optimized in the CD-MUSIC modeling.

### 3.5 Perspective

With the aim to resolve controversies regarding Pb immobilization mechanisms in environmental samples and to tackle modeling challenges associated with them, we investigated phosphate-enhanced lead immobilization on goethite, combining CD-MUSIC modeling with XAFS analysis and DFT calculations. Results show that the dominant mechanism is conditional. At relatively low PO_4_ concentrations, PO_4_ increases Pb immobilization on goethite via mainly electrostatic synergy; At relatively high PO_4_ concentrations, the formation of Pb-PO_4_ ternary complex becomes dominant at relatively low pH and low Pb concentrations, whereas at high pH and high Pb concentrations, Pb-PO_4_ precipitation plays a major role. The monodentate-O-sharing Pb-PO_4_ ternary complex is confirmed for the first time as the most favorable ternary structure on goethite. Simultaneous to the derivation of CD-MUSIC model parameters, this research also achieved a description of Pb-PO_4_ precipitation enhanced by goethite through probable heterogeneous nucleation by adjustment of log *K*_sp_ value.

The results imply that for heavily polluted soils, adding sufficient PO_4_ can significantly reduce the Pb availability as a result of Pb-PO_4_ precipitation. For lightly or nonpolluted soils, PO_4_ is also effective in mitigating Pb activity by electrostatic synergy and formation of ternary complex. Omitting ternary complexation would significantly underestimate Pb immobilization, which could potentially address the overestimation of soluble Pb in soils by e.g. multisurface models. The phenomenon of cation and anion co-adsorption on charged minerals in the environment is widespread, which is of significant importance for the simultaneous migration and release of nutrients and pollutants, and thus it is worthwhile to further explore more combinations of cations-(oxy)anions-minerals.^20^ This research provides a comprehensive modeling tool for PO_4_-mediated Pb immobilization on oxides, enabling precise predictions of electrostatic enhancement, ternary complexation, and surface precipitation of Pb. This advancement is significant for improving the accuracy of chemical speciation models in identifying the active interfaces that dominate Pb immobilization, thus enhancing the effectiveness of remediation strategies.

## Supporting Information Available

Synthesis and characterization of goethite and hydropyromorphite (HPM); charging behavior of goethite; CD-MUSIC model approach; protocol of FEFF-EXAFS analysis; DFT calculation; phosphate adsorption on goethite; verification of CD-MUSIC model parameters; simulations of other ion-phosphate ternary pairs; additional components of Pb L3-edge and P K-edge XANES spectra and LCF analysis; Pb speciation derived from CD-MUSIC model; additional components of EXAFS spectra and FEFF analysis; and surface charge properties derived from BVC and ESP analysis (PDF)
