# Supplementary material for: Quantitative Insights into Phosphate-Enhanced Lead Immobilization on Goethite
Source: Environ Sci Technol. 2024 Jun 24;58(26):11748–59. doi: 10.1021/acs.est.4c03927 (PMC11223472; doi:10.1021/acs.est.4c03927)
Supplement: Supplementary file 1 — es4c03927_si_001.pdf [file es4c03927_si_001.pdf]

Supporting Information

Quantitative Insights into Phosphate-Enhanced Lead Immobilization on Goethite

Wanli Lian,[†] Guanghui Yu,[§] Jie Ma,[†] Juan Xiong,[ǁ] Cuiyun Niu,[†] Ran Zhang,[†] Haijiao Xie[⊥] and Liping Weng[*,†,‡]

[†] *Key Laboratory for Environmental Factors Control of Agro-Product Quality Safety, Agro-Environmental Protection Institute, Ministry of Agriculture and Rural Affairs, Tianjin 300191, China*

[‡] *Department of Soil Quality, Wageningen University, P.O. Box 47, 6700AA Wageningen, The Netherlands*

[§] *Institute of Surface-Earth System Science, School of Earth System Science, Tianjin University, Tianjin 300072, China*

[ǁ] *Key Laboratory of Arable Land Conservation (Middle and Lower Reaches of Yangtze River), Ministry of Agriculture and Rural Affairs of the People's Republic of China, College of Resources and Environment, Huazhong Agricultural University, Wuhan 430070, China*

[⊥] *Hangzhou Yanqu Information Technology Co., Ltd, Hangzhou 310003, China*

[*] Corresponding author: Liping Weng

E-mail: liping.weng@wur.nl

Number of pages: 41

Number of Figures: 15

Number of Tables: 9

Content and Table & Figure Caption

Content	Page
**S1. Synthesis and Characterization of Materials**<br>◆ **Figure S1.** SEM image of synthesized goethite, and XRD patterns of the synthesized goethite and hydropyromorphite.<br>◆ **Figure S2.** pH-charging curve of synthesized goethite.	**S4-S6**
**S2. CD-MUSIC Model Approach**<br>◆ **Table S1.** Equilibrium constants of reactions at 25 °C for soluble complex formation and precipitation considered in the CD-MUSIC modeling.<br>◆ **Table S2.** Root mean square error (RMSE) between experimental and CD-MUSIC modeling results regarding Pb adsorption on goethite in this study.	**S7-S11**
**S3. Acquiring and Processing of Pb L3-Edge and P K-Edge XAFS Data**	**S12-S15**
**S4. DFT Calculations**<br>◆ **Figure S3.** DFT optimized Fe-cluster model and period goethite (110) face slab.	**S15-S20**
**S5. Phosphate Adsorption on Goethite in the Absence and Presence of Pb**<br>◆ **Figure S4.** Phosphate adsorption envelopes on goethite in the absence or presence of lead.	**S21**
**S6. Verification of CD-MUSIC Parameters of Pb Adsorption on Goethite**<br>◆ **Figure S5.** Comparison of experimental data of Pb adsorption to goethite from Wu et al. (2020) and Liang et al. (2021) with CD-MUSIC model calculations considering formation of Pb (hydrolyzed) bidentate surface species using parameters derived in this study.	**S22**
**S7. Modeling of Xie and Giammar (2007)'s Data Set**<br>◆ **Table S3.** CD-MUSIC model parameters employed to model Xie and Giammar's data set.<br>◆ **Table S4.** Experimental conditions and results from Xie and Giammar (2007), as well as the CD-MUSIC modeling based on the parameters derived in this study.	**S23-S24**
**S8. Comparative Analysis of Pb and Other Metal Ions in the Formation of Ternary Complexes with PO_4 on Iron (Hydr)oxides**<br>◆ **Figure S6.** Comparison of the ability of Pb and other metal cations to form ternary metal-PO_4-mineral complexes on goethite and ferrihydrite based on the CD-MUSIC model.	**S25-S26**

S9. Modeling Pb Immobilization on Goethite in the Presence of PO_4 Using Different Solubility Product of Hydropyromorphite **S27**

- **Figure S7.** CD-MUSIC modeling of Pb (300 μM) immobilization on goethite in the presence of 200 or 400 μM PO_4 using different solubility product (log K_{sp}) of HPM.

S10. Additional Results of Pb L3-Edge and P K-Edge XANES Spectra and LCF Analysis **S28-S31**

- **Figure S8.** LCF analysis results of Pb L3-edge spectra without and with considering Pb-PO_4 ternary complex.
- **Figure S9.** LCF fitting quality of Pb L3-edge spectra for the sample of 50 μM Pb + 400 μM PO_4 + goethite at pH 5, using different proportions of bidentate adsorbed Pb and precipitated Pb (HPM).
- **Table S5.** Correlations and RMSEs between Pb surface species quantified by Pb L3-edge XANES-LCF analysis and CD-MUSIC modeling.
- **Figure S10.** Normalized P K-edge XANES spectra of goethite with 400 μM PO_4 in the absence and presence of, along with the XANES spectra of HPM and the LCF analysis results.
- **Table S6.** Quantitative analysis of PO_4 species on goethite in the presence of Pb using LCF analysis of P K-edge XANES spectra and CD-MUSIC modeling.

S11. Quantifying Contribution of Different Mechanisms to Pb Immobilization on Goethite Induced by Phosphate Using the CD-MUSIC Model **S32**

- **Figure S11.** Contribution of different mechanisms to PO_4 induced immobilization of Pb on goethite as a function of pH.

S12. Additional Results of Pb L3-Edge EXAFS Analysis **S33-S36**

- **Figure S12.** Normalized k^2-weighted experimental Pb L3-edge EXAFS spectra and fitted value, with corresponding Fourier transformed magnitude and real parts of Fourier transform
- **Table S7.** EXAFS results of Pb complex with goethite in previous studies.
- **Table S8.** Coordination environment parameters of EXAFS results fitted by FEFF using Pb-Fe path instead of Pb-P path.
- **Figure S13.** Normalized k^2-weighted experimental Pb L3-edge EXAFS spectra and fitted value by using Pb-Fe path instead of Pb-P path as the second shell, with corresponding Fourier transformed magnitude and real parts of Fourier transform.
- **Figure S14.** Results from wavelet transform (WT) analysis of Pb EXAFS spectra.

S13. Analysis of Surface Charge Properties Using CD-MUSIC Model and DFT Calculations **S37-S39**

- **Table S9.** Electrostatic potential area from ESP analysis and charge distribution from BVC analysis based on the DFT calculation above the Fe-cluster of the interested surface complexes.
- **Figure S15.** Correlation of charge distribution properties derived from DFT calculations and from CD-MUSIC model.

References **S40-S44**

S1. Synthesis and Characterization of Materials

S1.1. Synthesis of Goethite

The synthesis of goethite (α-FeOOH) followed the method described by Hiemstra and Van Riemsdijk (1999), involving the following steps: A 2.5 M NaOH solution was added to a 0.50 M $Fe(NO_3)_3$ solution using a flow pump at a constant rate of 10 mL/min, while the suspension was stirred with an electric mixer. The pH of the solution was continuously monitored using a pH electrode, and the addition of NaOH solution was stopped when the pH reached 12.0. Then the suspension was aged in a 60 °C oven for 4 days. After aging, the supernatant was removed from the suspension and the precipitate was transferred to a dialysis bag and sealed with a clamp. The precipitate was then dialyzed in ultrapure water, and the water was replaced every 2 days. The electrical conductivity (EC) of the supernatant was measured during each water refreshment until the EC dropped to less than 10 μS/cm. Finally, the prepared goethite was transferred to a PET plastic bottle and stored at 4 °C. The specific surface area of goethite used in the current study was determined to be 80.9 m^2/g using BET-N_2 method. The morphology and purity of goethite was measured with SEM and XRD (Figure S1).

S1.2. Synthesis of Hydropyromorphite

The synthesis of pure lead hydropyromorphite (HPM, $Pb_5(PO_4)_3OH$) was carried out in accordance with the method reported by Zhu et al. (2015). The synthesis procedure involved the following steps: A 250 mL solution of 0.4 M $Pb(CH_3COO)_2 \cdot H_2O$ (lead acetate hydrate) was mixed with a 250 mL buffer solution of 4.4 M CH_3COONH_4 (ammonium acetate) in a 1 L polypropylene vessel. Subsequently, 500 mL of 0.12 M $NH_4H_2PO_4$ (ammonium dihydrogen phosphate) solution was rapidly added to the vessel under stirring, resulting in the formation of a white suspension. The suspension was then adjusted to pH 7.5 using NH_4OH (ammonium hydroxide) solution

and stirred for 10 minutes at room temperature. The suspension was aged at 100 °C for 48 hours to promote further reaction and precipitation of the desired product. After aging, the resulting precipitate was allowed to settle, and was then thoroughly washed with ultrapure water to remove impurities. Finally, the washed precipitate was dried in an oven at 70 °C for 16 hours to obtain the pure HPM product.

S1.3. Acid-base Titration of Goethite

The titration was conducted under N_2 atmosphere using 0.05 M NaOH as the base and 0.05 M HNO_3 as the acid. A suspension of goethite (5 g/L) in 0.01 M $NaNO_3$ was prepared, and the titration was initiated with 50 mL of the suspension. Prior to titration, the suspension was adjusted to pH 4.0, and pH electrode readings and drift were monitored until the drift was below 0.2 mV/min (or 0.01 pH unit/min) for 60 s. Successive base titrations (up to pH 10.0) and acid titrations (back to pH 4.0) were performed at three different $NaNO_3$ concentrations (0.01, 0.05, and 0.1 M). The theoretical blank was calibrated using the extended Davies equation for activity coefficients calculations by comparing the experimental blank titrations at corresponding ionic strength values. The relative charge density of goethite was determined by subtracting the theoretical blank titration consumption from the sample titration consumption, as reported by Antelo et al. (2015), to express the relative charge density as a function of pH. A pH-static titration with $NaNO_3$ at pH 5.0 was performed to obtain the relative change of charge density between the three $NaNO_3$ concentrations at pH 5.0, and thus the absolute charge density curves over the whole pH range can be plotted together. The common intersection point of the curves at the three $NaNO_3$

concentrations was determined as the original point of zero charge (PZC), which was then used to construct the absolute charge density curve.

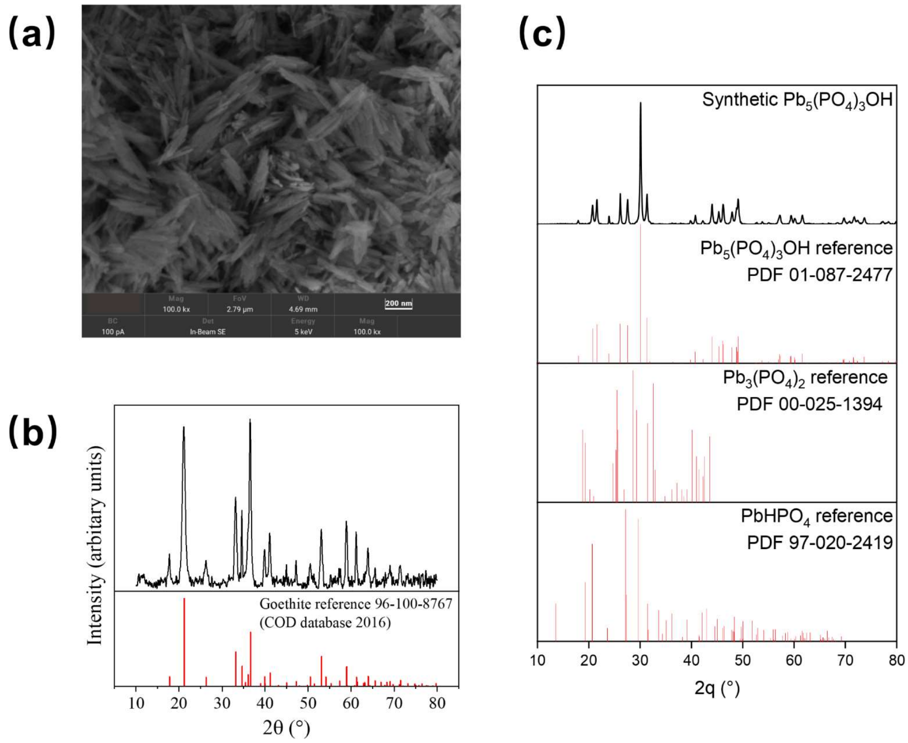

Figure S1. SEM image of synthesized goethite (a), and XRD patterns of the synthesized goethite (b) and hydropyromorphite (c).

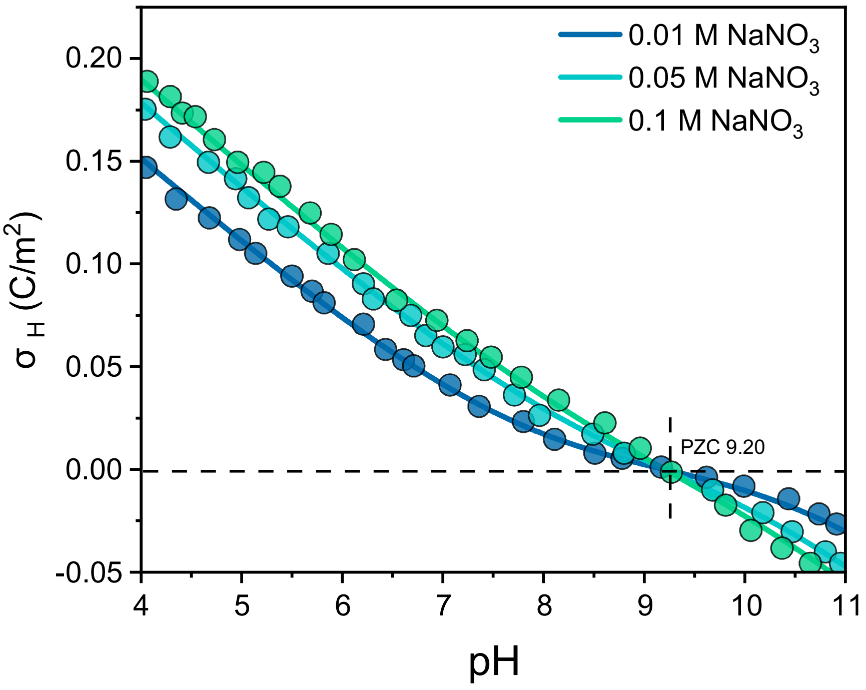

Figure S2. pH-charging curve of synthesized goethite. Dots are experimental data, lines are CD-MUSIC modeling.

S2. CD-MUSIC Model Approach

S2.1. Proton Binding and Ion Pairs:

The CD-MUSIC model used in this study considers two types of reactive groups on the goethite surface: $\equiv Fe_3OH^{-0.5}$ and $\equiv FeOH^{-0.5}$, with respective site densities of 2.7 and 3.45 sites/nm^2 (Hiemstra and Van Riemsdijk, 2006). Spectroscopic studies suggest that the (110) face is dominant on goethite, while the (021) face accounts for only 5–20% of the surface area (Venema et al., 1996; Villalobos and Pérez-Gallegos, 2008). In this study, it was assumed that $\equiv FeOH_L^{-0.5}$ on the (110) face were low-affinity sites for Pb adsorption, accounting for 85% (2.93 sites/nm^2) of the total $\equiv FeOH^{-0.5}$ sites, while $\equiv FeOH_H^{-0.5}$ on the (021) face were high-affinity sites, accounting for the remaining 15% (0.52 sites/nm^2) of the total $\equiv FeOH^{-0.5}$ sites. The extended Stern model was used to describe the electrostatic structure on goethite, which is represented by three electrostatic planes, namely 0-plane, 1-plane, and 2-plane. The inner and outer Stern layer capacitances (C_1, C_2) are 0.85 and 0.75 F/m^2, respectively, adopted from Hiemstra and Van Riemsdijk (2006). The CD-MUSIC model uses a proton binding affinity constant for all sites, which is equal to the PZC (point of zero charge). The PZC of goethite was determined to be 9.20 in this study with acid-base titration (Figure S2). Electrolyte ions of Na^+ and NO_3^- form ion pairs with all sites with equal log K values taken from Hiemstra and Van Riemsdijk (2006). The aforementioned CD-MUSIC parameters accurately described the surface charging behavior of goethite in this study (Figure S2).

S2.2. Pb and PO4 Adsorption in Mono-component Systems:

In the CD-MUSIC modeling of goethite, $\equiv FeOH^{-0.5}$ is generally treated as the only reactive site complexing Pb and PO_4. For PO_4, both monodentate ($\equiv FeOPOOH^{-1.5}$) and bidentate ($\equiv Fe_2O_2PO_2^{-2}$) inner-sphere complexes were considered (Rahnemaie et al.,

2007a). The charge distribution (CD) values (Δz_0, Δz_1) of these PO_4 complexes have been studied in detail previously with DFT calculation, which were adopted in this study (Rahnemaie et al., 2007a). For Pb, a bidentate ($\equiv(FeOH)_2Pb^+$) inner-sphere complex was considered. Adsorption of Pb on the (021) face of goethite occurs through an edge-sharing complex ($\equiv(FeOH_H)_2Pb^+$) with high-affinity, while on the (110) face, it forms a corner-sharing complex ($\equiv(FeOH_L)_2Pb^+$) with low-affinity, as confirmed by the current and previous EXAFS and modeling studies (Elzinga et al., 2001; Leung and Criscenti, 2017; Ostergren et al., 2000b, 2000a, 1999; Wu et al., 2020).

To describe the adsorption of Pb at high pH, the hydrolysis species of Pb ($\equiv(FeOH)_2PbOH^0$) was also taken into account. For simplicity, the coordination of Pb with $\equiv Fe_2OH^{-0.5}$ and $\equiv Fe_3OH^{-0.5}$ sites was not taken into account in this study. Although the coordination of Pb with these two surface functional groups has been verified using EXAFS spectroscopy and molecular dynamics simulations (Leung and Criscenti, 2017; Ostergren et al., 2000b), calculations from the CD-MUSIC model of Liang et al. (2021) found that the complexation of these two sites with Pb had a negligible contribution, accounting for less than 10%, to Pb adsorption.

S2.3. Pb-PO_4 Ternary Complexation:

For Pb-PO_4 co-existing systems, one type of O-sharing ternary Pb-PO_4 complex ($\equiv(FeO)_2HPbPO_3H^0$) was considered in CD-MUSIC modeling (Model A, based on EXAFS and DFT analysis of this study and the literature (Tiberg et al., 2013), see text). In order to reveal the necessity of including such ternary surface complex, we also fitted the experimental data to the model while neglecting the ternary surface complex (Model B). The optimization of the CD-MUSIC parameters of such ternary complex will be discussed below.

It should be noted that log K values for bidentate Pb surface species and the Pb-PO_4 ternary surface complex differed for high and low-affinity sites. Conversely, proton affinity constants, ion pair formation constants, and binding constants of PO_4 to goethite were the same for both high and low-affinity sites (Table 1).

S2.4. Precipitation:

In modeling Pb precipitation, the mineral hydropyromorphite (HPM, $Pb_5(PO_4)_3OH$) was considered due to its low solubility compared to other minerals in bulk solution such as $Pb_3(PO_4)_2$, $PbHPO_4$, and $Pb(OH)_2$ (log K_{sp} values of $Pb(OH)_2$, $Pb_3(PO_4)_2$, $PbHPO_4$, and $Pb_5(PO_4)_3OH$ are −19.84, −44.36, −23.80, and −80.77, respectively). In this study, the solubility product (log K_{sp}) of HPM was optimized, resulting in a value of −82.02, which is lower than those reported for HPM in the bulk solution (−80.77–−76.79) (Lindsay, 1979; Zhu et al., 2015). In Figure S7, modeling results of Pb immobilization on goethite for the treatment of 300 μM Pb and 400 μM PO_4 are compared when using the log K_{sp} value for HPM derived in this study and reported in the literature. The precipitation could have been enhanced by goethite through heterogeneous nucleation or formation of solid solution (Karthikeyan and Elliott, 1999; Lützenkirchen and Behra, 1995).

While $Pb(OH)_2$, $Pb_3(PO_4)_2$, and $PbHPO_4$ were considered in the calculations, their log K_{sp} values were not optimized. This was due to the fact that these minerals did not form in our experimental system during subsequent model calculations, as well as to reduce the complexity of the calculations. In fact, this omission is reasonable from a thermodynamic perspective, as more soluble precipitates will slowly transform into less soluble ones (Manecki et al., 2020). Furthermore, according to our subsequent Pb L3-edge XANES spectra, the spectral characteristics of the precipitates in the samples are consistent with HPM (see text). In addition, according to the quantitative results of the

LCF of Pb L3-edge and P K-edge, the ratio of P to Pb atoms in the precipitates is close to 0.6 in HPM, all of which strongly confirm that HPM is the primary precipitate (see text).

S2.5. Geochemical Code:

The calculation and parameter optimization were carried out using ECOSAT 4.9 and FIT software. Solution reactions and thermodynamic equilibrium constants used in the modeling are provided in Table S1. Activity coefficients for each aqueous species were calculated using the Davies equation.

S2.6. Optimization of CD-MUSIC Model Parameters:

Based on PO_4 adsorption data in the absence of Pb, the log *K* values for PO_4 adsorption ($\equiv FeOPOOH^{-1.5}$ and $\equiv Fe_2O_2PO_2^{-2}$) on goethite were optimized, using CD values taken from Rahnemaie et al. (2007).

Using data of Pb adsorption in the absence of PO_4, log *K* values for the bidentate ($\equiv(FeOH)_2Pb^+$), hydrolyzed bidentate ($\equiv(FeOH)_2PbOH^0$) surface species were optimized, while keeping the CD values the same as in Weng et al. (2001) for all bidentate Pb species.

By keeping the above-mentioned parameters as those fitted, the CD and log *K* values for the ternary Pb-PO_4 surface complex were then fitted using the batch adsorption experiment data of Pb in the presence of PO_4 in which no precipitation was apparently formed (derived from XANES-LCF results) (pH 2.8–5, 10 and 50 μM Pb co-adsorbed with 200 and 400 μM PO_4 on goethite under 10 mM $NaNO_3$). For this ternary complex, differentiation was also made for the high and low affinity site in terms of log *K*.

In an effort to ascertain the precision of the model, a computation of the root mean square error (RMSE) was performed. This was done by comparing the model predicted

values of Pb adsorption percentages with the corresponding experimental data across various treatments. The resulting RMSE values have been tabulated in Table S2.

Table S1. Equilibrium constants of reactions at 25 °C for soluble complex formation and precipitation considered in the CD-MUSIC modeling.

Species	Reaction	log *K*
Solution complexation (I = 0)		
$PbOH^+$	$Pb^{2+} + H_2O \leftrightarrow PbOH^+ + H^+$	6.30[a]
$Pb(OH)_2$	$Pb^{2+} + 2H_2O \leftrightarrow Pb(OH)_2^0 + 2H^+$	10.2[a]
$PbNO_3^+$	$Pb^{2+} + NO_3^- \leftrightarrow PbNO_3^+$	1.17[a]
$Pb(NO_3)_2^0$	$Pb^{2+} + 2NO_3^- \leftrightarrow Pb(NO_3)_2^0$	1.40[a]
$PbHPO4^0$	$Pb^{2+} + H^+ + PO_4^{3-} \leftrightarrow PbHPO_4^0$	15.45[a]
$PbH_2PO_4^+$	$Pb^{2+} + 2H^+ + PO_4^{3-} \leftrightarrow PbH_2PO_4^+$	21.05[a]
HPO_4^{2-}	$PO_4^{3-} + H^+ \leftrightarrow HPO_4^{2-}$	12.35[a]
$H_2PO_4^-$	$PO_4^{3-} + 2H^+ \leftrightarrow H_2PO_4^-$	19.55[a]
$H_3PO_4^0$	$PO_4^{3-} + 3H^+ \leftrightarrow H_3PO_4^0$	21.70[a]
$NaHPO_4^-$	$PO_4^{3-} + H^+ + Na^+ \leftrightarrow NaHPO_4^-$	13.40[b]
$Na(PO_4)^{2-}$	$PO_4^{3-} + Na^+ \leftrightarrow Na(PO_4)^{2-}$	2.05[b]
Precipitation		log K_{sp}
$Pb(OH)_2(s)$	$Pb(OH)_2(s) \leftrightarrow Pb^{2+} + 2OH^-$	−19.84[a]
$PbHPO_4(s)$	$PbHPO_4(s) \leftrightarrow Pb^{2+} + H^+ + PO_4^{3-}$	−23.80[a]
$Pb_3(PO_4)_2(s)$	$Pb_3(PO_4)_2(s) \leftrightarrow 3Pb^{2+} + 2PO_4^{3-}$	−44.36[a]
$Pb_5(PO_4)_3OH(s)$	$Pb_5(PO_4)_3OH(s) \leftrightarrow 5Pb^{2+} + 3PO_4^{3-} + OH^-$	−82.02[c]

a. From Lindsay (1979); b. From Rahnemaie et al. (2007); c. Optimized in this study.

Table S2. Root mean square error (RMSE) between experimental and CD-MUSIC modeling results regarding Pb adsorption on goethite in this study.

Pb	Without PO_4	With 200 μM PO_4		With 400 μM PO_4	
(μM)	Model	Model A[a]	Model B[b]	Model A	Model B
10	4.32%	1.65%	7.27%	1.78%	28.94%
50	2.90%	2.91%	3.67%	2.71%	22.83%
100	3.04%	2.58%	3.42%	2.01%	13.37%
300	2.77%	3.49%	3.44%	2.26%	7.99%
Overall	3.32%	2.74%	4.74%	2.22%	20.01%

a. Calculations considering Pb-PO_4 ternary complex. b. Calculations omitting Pb-PO_4 ternary complex.

S3. Acquisition and Processing of Pb L3-Edge and P K-Edge XAFS Data

S3.1. Details in XAFS experiment

XAFS analysis was employed to detect Pb and P surface species on goethite. Selected samples from adsorption experiments (50–300 μM Pb and 200–400 μM PO_4 at pH 5 or 7 in 10 mM $NaNO_3$) as well as HPM were subjected to Pb L3-edge and P K-edge spectra collection. Post-experiment, the solids were centrifuged again, freeze-dried, ground uniformly, and sealed. To improve the signal-to-noise ratio, samples with low concentrations were compressed into 6 mm-diameter pellets (~75 mg). HPM and two samples containing 300 μM Pb and either 200 or 400 μM PO_4 at pH 5 were diluted using boron nitride (BN) to mitigate self-absorption. The HPM was diluted approximately 15-fold, as calculated by the Hephaestus software (Ravel and Newville, 2005). For the sample containing 300 μM Pb and PO_4, the dilution was 1.5 and 3-fold at respectively 200 and 400 μM PO_4. All spectra were collected at room temperature, with multiple spectra acquired to reduce noise.

Pb L3-edge (13035 eV) spectra were obtained with 14W beamline at Shanghai Synchrotron Radiation Facility (SSRF) using a Si (111) monochromator. The spectra were collected in fluorescence mode using a 32-element detector, with a Ge filter to eliminate Fe fluorescence disruptions. Spectra of HPM were collected in transmission mode. E_0 was calibrated with the maximum of the first derivative of spectra of Pb foil. The energy scan range for all Pb L3-edge spectra was −200–−20 eV (relative to the Pb L3-edge) in 4 eV steps; −20–50 eV in 0.5 eV steps; 50–200 eV in 2 eV steps; 200–400 eV in 3 eV steps; 400–500 eV in 4 eV steps. The electron storage ring was operated at a voltage of 3.5 GeV and a current of 250 mA. For linear combination fitting (LCF), an energy scan range of −25 to 75 eV was utilized, comprising 157 data points. The

normalized intensity (E) within the −25 to 75 eV range exhibited a relative standard deviation of 2.3% to 4.8% across different spectra of parallel/or same sample(s).

P K-edge (2145.5 eV) XANES spectra were collected using beamline 4B7A at Beijing Synchrotron Radiation Facility (BSRF) with a double crystal monochromator and a silicon drifted detector. Spectra were collected at partial fluorescence mode. E_0 was calibrated with the maximum of the first derivative of the spectra of black phosphorus powder. The chamber pressure was maintained at 10^{-6} Torr and in N_2/He gas environment during the measurement. The step size was 1 eV in the pre-edge region (2130–2140 eV), 0.2 eV in the edge jump region (2140–2175 eV), and 0.5 eV in the post-edge region (2175–2200 eV). The working voltage of the electron storage ring was 2.5 GeV, with a current of 150–250 mA. The normalized intensity (E) within the −10 to 40 eV range (200 data points) showed a relative standard deviation of 1.8% to 3.2% among different spectra of parallel/or same sample(s).

No self-absorption correction was performed in further data processing because transmission mode was used to analyze HPM diluted by BN in which concentrations of both Pb and P were relatively high, while other samples measured in fluorescence mode contain relatively low concentrations (0.79%–1.58% wt for Pb; 0.59%–0.88% wt for P). Noticeable self-absorption could occur at concentrations above 2% wt, so no or negligible self-absorption occurred. This was further confirmed by monitoring the coefficients related to self-absorption in real time during the analysis. For example, the dead time parameter of the signal was always in the normal range (below 5) for P K-edge XANES analysis, and the signal-to-noise ratios of XAFS spectra were always in a reasonable range for Pb L3-edge analysis.

S3.2. Processing of Pb L3-Edge EXAFS Data

The EXAFS data were analyzed using the Demeter suite of programs, including Athena and Artemis, which are widely used in the field of XAFS data analysis (Ravel and Newville, 2005). The raw intensity data were firstly converted to μ(*E*), and spectra from multiple tests were merged to obtain higher quality spectra for further analysis. The EXAFS signal, denoted as χ(*k*), where *k* represents the photoelectron wavenumber, was then processed using the AUTOBK algorithm to subtract the background.

The χ(*k*) spectra were weighted by k^2. These oscillations in χ(*k*) were characterized using the EXAFS equation, which involves the photoelectron backscattering amplitude [*f*(*k*)], phase shift [$\delta(k)$], number of neighboring atoms (*N*), distance to the neighboring atom (*R*), and the Debye-Waller factor (σ^2) representing the disorder in the neighbor distance:

$$\chi(k) = \sum_j \frac{N_j f_j(k) e^{-2k^2\sigma_j^2}}{kR_j^2} \sin\left[2kR_j + \delta_j(k)\right] \qquad \text{(Eq. S-1)}$$

The k^2 weighted EXAFS spectra in *k*-space (Å^{-1}) were Fourier transformed (FT) into R-space (Å) for further analysis. The theoretical phase-shift [$\delta(k)$] and amplitude functions for single-scattering [*f*(*k*)] were calculated using the *ab initio* computer code FEFF6, based on atomic clusters generated from the crystal structures of the samples under investigation, which included PbO (Leciejewicz, 1961) with partial Fe substitute and HPM (Barinova et al., 1998).

Several fitting parameters were used during the analysis, including the many-body amplitude reduction factor (S_0^2), which was established as 0.8 based on fitting to the Pb foil standard. The parameter ΔE_0, representing the difference between the threshold energy and the FEFF phase shift function, was fixed during fitting but varied slightly from sample to sample. The σ^2 for different shells of each sample was set to be consistent, and was allowed to float during the fitting process. Interatomic distances (*R*) and coordination numbers (CN) were also allowed to float during fitting.

The value of rbkg, which represents the threshold below which the AUTOBK algorithm removes Fourier components, was set to 1.0 for all samples. The quality of the fits was evaluated using the R-factor, a goodness-of-fit parameter, calculated as the sum of squared differences between the experimental χ data and the fitted χ values, divided by the sum of squared χ data. The fits were considered as good when the R-factor was less than 0.05, indicating a high level of agreement between the experimental data and the fitted model.

The wtEXAFS software package (Zhihang, 2023) was employed to perform wavelet transformation on the k^2-weighted EXAFS data using the Morlet wavelet transformation method. For all samples, the parameters η and σ were set to 5 and 1, respectively, and the range of k used was 3–8 Å^{-1}.

S4. DFT Calculations

S4.1. Cluster DFT Calculations

All cluster Density Functional Theory (DFT) calculations using the Gaussian 16 software suite (Frisch et al., 2016). The PBE0 functional was employed for all cluster DFT calculations (Adamo and Barone, 1999), supplemented with the D3BJ dispersion correction (Grimme et al., 2010). The choice of PBE0 as the functional was based on extensive testing, which demonstrated its suitability for transition metal compounds (Dohm et al., 2018; Maurer et al., 2021). The PBE0 has also been successfully applied to simulate ion adsorption on binuclear Fe clusters, yielding reliable results (Paul et al., 2007). In this study, the cluster DFT calculated Pb bidentate complex, using PBE0, yielded a Pb–Fe distance of 3.87 Å. This is in close agreement with the 3.88–4.00 Å range reported in the literature based on EXAFS measurements (Elzinga et al., 2001;

Ostergren et al., 2000a, 2000b, 1999). The calculated Pb–P and Pb–Fe distance of ternary complex are 3.95 and 3.57 Å, respectively. These distances in EXAFS are 3.97 and 3.55 Å. The average difference of cluster DFT and EXAFS is 0.06 Å. Additionally, calculations were also performed using the B3LYP and BP86 functional, which yielded a Pb–Fe distance of 3.85 and 3.81 Å, respectively, demonstrating the results of PBE0 density functional are more access to EXAFS.

The binuclear Fe(III) cluster was used, with the multiplicity set to a high-spin ferromagnetic state. This binuclear iron cluster provides a simplified modeling approach to simulate the reactivity of iron oxide surfaces. As demonstrated by Paul et al. (2007), this modeling method is considered sufficient to reproduce the local binding structures of periodic iron oxides' surfaces. It has been applied multiple times and has been shown to yield valuable results of surface complex structure on goethite (Goli et al., 2011; Hiemstra et al., 2007; Hiemstra and Van Riemsdijk, 2006; Rahnemaie et al., 2007b, 2007a; Xu et al., 2016; Yan and Jing, 2018) and ferrihydrite (Ma et al., 2023; Mendez and Hiemstra, 2020; Van Eynde et al., 2022).

For geometry optimization and frequency calculations, the 6-31+G(d) basis set was utilized for O, H and P, while the SDD was used for Fe and Pb (Hariharan and Pople, 1973; Schwerdtfeger et al., 1989). The SMD solvation model was implemented to account for the solvation effect of water in all calculations (Marenich et al., 2009).

The cluster DFT calculations included the structural optimization of the binuclear iron cluster, bidentate adsorption of Pb, monodentate and bidentate adsorption of phosphate, ternary complexation reactions of lead-bridged, phosphate-bridged, and monodentate-oxygen-sharing ternary complexes. The stoichiometry of the three different types of ternary complexation reactions is identical, which involves two

surface sites, two H, one Pb and one PO_4. After the optimization, the frequency analysis of all structures shows that there is no imaginary frequency.

The cluster DFT optimized geometries were used to derive the CD coefficients (Δz_0 and Δz_1) of the adsorbed complexes of interest in this study. The optimized geometries were interpreted with the bond valence concept (BVC, Brown and Altermatt, 1985), which relates bond length (R) to a bond valence (v) according to:

$$v = \exp\left(-\frac{R - R_0}{B}\right) \qquad \text{(Eq. S-2)}$$

where, B is an empirical constant (B = 37 pm), and R_o (pm) is a reference distance whose value is chosen in such a way that the sum of v is equal to the formal valence of the adsorbing ion. Table S9 presents the values of R (pm) and the corresponding values of v (v.u.) for Pb or P in surface species of interest.

From the bond valence (v) values, the ionic charge distribution values on goethite (n_0 and n_1) were calculated. In addition to these values, the final CD coefficients (Δz_0, Δz_1) also include the charge of the protons involved in the formation reactions (nH_0, nH_1) and a correction term ($\pm \varphi_m \Lambda_0$) for interfacial water dipole orientation, where φ_m is a constant (0.17 ± 0.02) and Λ_0 is the change of charge relative to that of the reference state from which the reaction is defined. Details about the calculations are explained by Hiemstra and van Riemsdijk (2006). The final CD coefficients for all surface species of interest are summarized in Table S9.

The electrostatic potential (ESP) profiles based on wavefunction calculated by cluster DFT were analyzed using Multiwfn (Lu and Chen, 2012). The visualized version was their ESP surfaces (see Figure 3 in text) which were printed by VMD (Humphrey et al., 1996).

S4.2. Periodic DFT Calculations

The periodic DFT computational study was conducted using the spin-polarized DFT approach (Hohenberg and Kohn, 1964; Kohn and Sham, 1965), utilizing the Vienna ab initio simulation package (VASP). This package employs plane-wave basis sets in conjunction with the projector augmented-wave method (Blöchl, 1994; Hafner, 2008; Kresse and Furthmüller, 1996). The exchange-correlation potential was addressed using a generalized gradient approximation (GGA) following the Perdew-Burke-Ernzerhof (PBE) parametrization (Perdew et al., 1996). The PBE functional has successful used to modeling ion adsorbed on goethite periodic surface for several times (Leung and Criscenti, 2017; Paul et al., 2007; Wang et al., 2024). To rectify the limitations of GGA, we incorporated the GGA + U method in our calculations. The effective Hubbard U value was assigned as 6.0 eV for the Fe 3d orbital, as suggested by prior research (Fabiano et al., 2010; Fuente et al., 2013). Additionally, we applied Grimme's DFT-D3 model for the van der Waals correction (Grimme et al., 2010). We set the energy cutoff at 500 eV and sampled the Brillouin-zone integration with a single Γ point (1 × 1 × 1). The PAW pseudopotentials were used to describe the valence electrons of elements, where the valence electrons of Fe, O, H, P, and Pb were $3d^7 4s^1$, $2s^2 2p^4$, $1s^1$, $2s^2 2p^3$, and $5d^{10} 6s^2 6p^2$, respectively. The surface dipole correction was utilized in the surface calculations. In the structural optimization calculation, the conjugate gradient algorithm (CG) was used to relax all atoms, and the maximum convergence criterion of the geometry optimization was 0.05 $eV \cdot Å^{-1}$, with an energy convergence standard of 10^{-5} eV. The optimized bulk cell parameters after cell optimization are a = 4.556 Å, b = 9.967 Å, c = 3.022 Å, $\alpha = \beta = \gamma = 90°$. The (110) face of completely hydroxylated α-FeOOH consists of two α-FeOOH atomic layers with a total of 152 atoms, including 32 Fe, 48 H and 72 O atoms. A 15 Å vacuum layer was

created to ignore the interaction between surfaces. The adsorption energy, denoted as E_{ads}, can be calculated as follows:

$$E_{ads} = E_{*+\mathrm{mol}} + 2E_{\mathrm{H2O}} - E_{*} - E_{\mathrm{mol}} \qquad \text{(Eq. S-3)}$$

where $E_{*+\mathrm{mol}}$ represents the energy of the goethite (110) surface with the adsorbate, E_{*} is the energy of a hydrated surface, and E_{H2O} and E_{mol} denote the energy of an H_2O molecule and the adsorbate in a vacuum, respectively. Due to the bidentate adsorption of the 6-coordinated Pb, two H_2O molecules are replaced to form a 4-coordinated H_2O Pb (the total coordination number remains 6). In order to maintain the electrical neutrality of the system, additional hydrogen ion was added to the surface while adsorbing ions when necessary.

All the surface species structures to be calculated in the periodic DFT are the same as cluster DFT. The visualized version of optimized structures printed by VESTA (Momma and Izumi, 2011). For lead adsorption structures using periodic DFT calculations, the Pb–Fe distance in bidentate Pb complex was found to be 3.90 Å, which is consistent with the cluster DFT result of 3.87 Å and closely aligns with the EXAFS measurement of 4.00 Å. For the monodentate Pb–PO_4 oxygen-sharing complex, the Pb–P and Pb–Fe distances were calculated to be 3.51 and 4.11 Å respectively. These results are in agreement with the cluster DFT distances of 3.57 and 3.95 Å, and the EXAFS measurements of 3.55 and 3.97 Å respectively. In the phosphorus-bridged ternary complex, the Pb–P and Pb–Fe distances were 2.88 and 6.03 Å, compared to 3.02 and 5.80 Å in cluster DFT calculations. For the Pb-bridged ternary complex, the distances were 3.87 Å for Pb–P and 3.90 Å for Pb–Fe, which are close to the cluster

DFT results of 3.62 and 3.92 Å. It is obviously that both periodic DFT and cluster DFT provide fundamentally similar descriptions of bond lengths. The key Pb surface complexes' bond lengths calculated by both cluster and periodic DFT, as well as measured by EXAFS, the average differences less than 0.1 Å. This underscores the close concordance of the two DFT calculations with EXAFS, affirming their precision. Further discussion on this topic is no longer discussed in the main text.

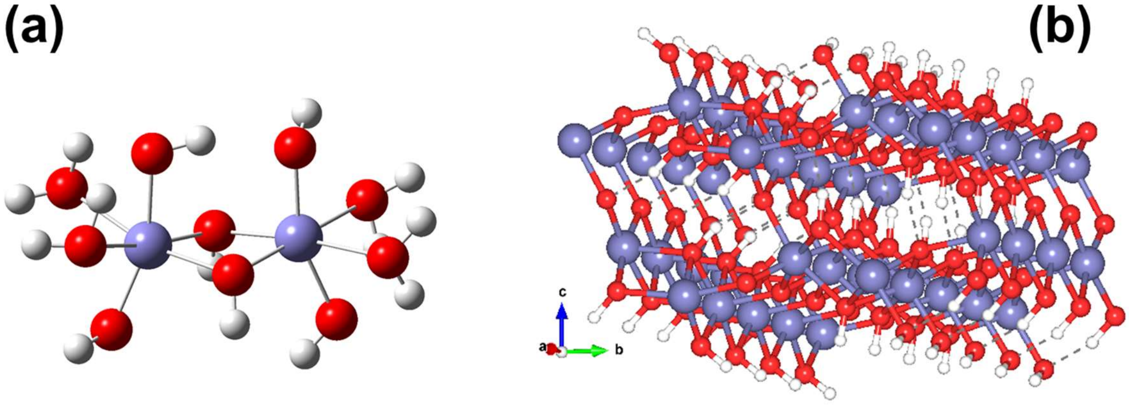

Figure S3. DFT optimized Fe-cluster model (a) and period goethite (110) face slab (b). The color of light blue, red and white spheres denote as Fe, O and H atom, respectively.

S5. Phosphate Adsorption on Goethite in the Absence and Presence of Pb

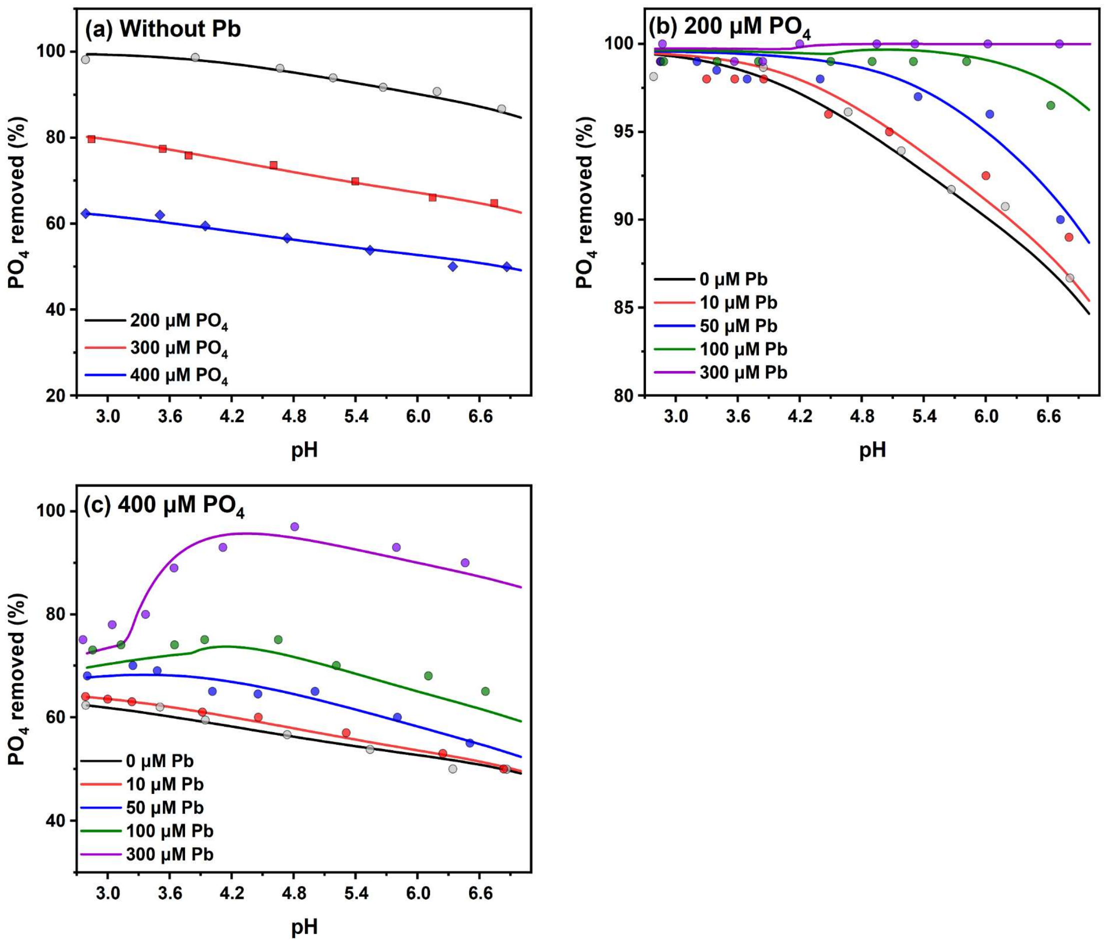

Figure S4. Phosphate adsorption envelopes on goethite in the absence or presence of lead. (a). Phosphate (200, 300, and 400 µM) adsorption in the absence of Pb. (b) & (c). Phosphate (200 and 400 µM) adsorption in the presence of 10–300 µM Pb. Goethite: 1.3 g/L (105 m^2/L). Background electrolyte: 10 mM $NaNO_3$. Symbols are experimental results, lines are CD-MUSIC modeling (Model A).

S6. Verification of CD-MUSIC Parameters of Pb Adsorption on Goethite

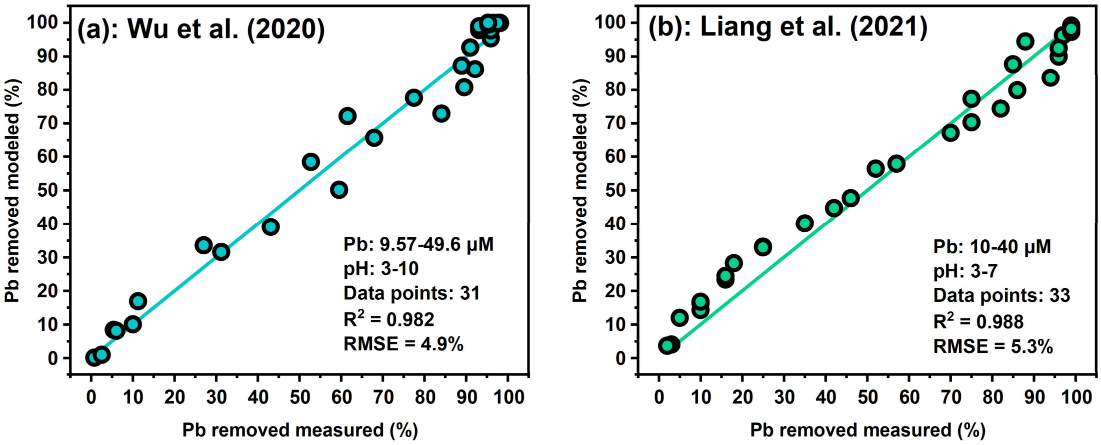

Figure S5. Comparison of experimental data of Pb adsorption to goethite from Wu et al. (2020) (a) and Liang et al. (2021) (b) with CD-MUSIC model calculations considering formation of Pb (hydrolyzed) bidentate surface species using parameters derived in this study (Table 1). Circles are modeling results and the line is 1 to 1 line. In Wu et al. (2020): goethite: 1.0 g/L, specific surface area: 74.49 m^2/g, background: 10 mM $NaNO_3$; In Liang et al. (2021): goethite: 1.0 g/L, specific surface area: 63.12 m^2/g, background: 10 mM KNO_3. The site density, specific surface area, capacitance, PZC, ion pair parameters followed the literature, while the reaction parameters of Pb followed the present study.

S7. Modeling of Xie and Giammar (2007)'s Data Set

Table S3. CD-MUSIC model parameters employed to model Xie and Giammar's data set.

surface complexation reactions	CD-values			log *K*
	Δz_0	Δz_1	Δz_2	
proton binding reactions				
$FeOH^{-0.5} + H^{+} \leftrightarrow FeOH_2^{+0.5}$	1	0	0	9.20[a]
$Fe_3O^{-0.5} + H^{+} \leftrightarrow Fe_3OH^{+0.5}$	1	0	0	9.20[a]
ion pairs				
$FeOH^{-0.5} + Na^{+} \leftrightarrow FeOHNa^{+0.5}$	0	1	0	−0.60[a]
$Fe_3O^{-0.5} + Na^{+} \leftrightarrow Fe_3ONa^{+0.5}$	0	1	0	−0.60[a]
$FeOH^{-0.5} + H^{+} + NO_3^{-} \leftrightarrow FeOH_2NO_3^{-0.5}$	1	−1	0	8.62[a]
$Fe_3O^{-0.5} + H^{+} + NO_3^{-} \leftrightarrow Fe_3OHNO_3^{-0.5}$	1	−1	0	8.62[a]
inner-sphere complexation				
$2FeOH^{-0.5} + PO_4^{3-} + 2H^{+} \leftrightarrow Fe_2O_2PO_2^{-2} + 2H_2O$	0.46	−1.46	0	29.31[a]
$FeOH^{-0.5} + PO_4^{3-} + 2H^{+} \leftrightarrow FeOPO_2OH^{-1.5} + H_2O$	0.28	−1.28	0	27.35[a]
$2FeOH_L^{-0.5} + Pb^{2+} \leftrightarrow (FeOH_L)_2Pb^{+}$	1.15	0.85	0	11.54[b]
$2FeOH_H^{-0.5} + Pb^{2+} \leftrightarrow (FeOH_H)_2Pb^{+}$	1.15	0.85	0	12.45[a]
$2FeOH_L^{-0.5} + Pb^{2+} + H_2O \leftrightarrow (FeOH_L)_2PbOH^{0} + H^{+}$	1.15	−0.15	0	2.10[a]
$2FeOH_H^{-0.5} + Pb^{2+} + H_2O \leftrightarrow (FeOH_H)_2PbOH^{0} + H^{+}$	1.15	−0.15	0	3.62[a]
$2FeOH_L^{-0.5} + Pb^{2+} + PO_4^{3-} + 2H^{+} \leftrightarrow (FeO_L)_2HPbPO_3H^{0} + H_2O$	0.60	0.40	0	32.25[b]
$2FeOH_H^{-0.5} + Pb^{2+} + PO_4^{3-} + 2H^{+} \leftrightarrow (FeO_H)_2HPbPO_3H^{0} + H_2O$	0.60	0.40	0	33.35[b]

a. Same as in Table 1. b. Minor adjustment to fit Xie and Giammar (2007)'s dataset.

In the study conducted by Xie and Giammar (2007), a concentration of 50 g/L of goethite-coated sand was used. The goethite content in this mixture was 0.2% wt (in Fe). The Fe content in the goethite was found to be 62.8% wt. This translates to an actual goethite concentration of 0.16 g/L for current CD-MUSIC modeling. The specific surface area of the goethite in Xie and Giammar (2007) was determined to be 33.6 m^2/g. The background is 15 mM $NaNO_3$ in their experiment. In Xie and Giammar (2007), the 1-pK model was employed. In our CD-MUSIC modeling, parameters of site density, capacity for inner and outer layers (C_1, C_2), PZC, and ion pair reactions were all kept the same as in modeling data in current study (Table 1 in text), whereas small adjustment was made for the log *K* values of Pb complexes. An additional mineral ($Pb_5(PO_4)_3Cl$) was considered (because Cl was present in their system, and HPM was also considered), with the log K_{sp} value of −84.43 following Xie and Giammar (2007).

Table S4. Experimental conditions and results from Xie and Giammar (2007), as well as the CD-MUSIC modeling based on the parameters derived in this study. The green shadowed row represents the sample with precipitation.

pH	total Pb (M)	total PO_4 (M)	total Cl (M)	Pb removal (%)		precipitate (%)	bidentate (%)	ternary (%)
				experiment	modeling			
4	5.00E−07	0.00E+00	0.00E+00	6.7	5.8	0.00	100.0	0.0
5	5.00E−07	0.00E+00	0.00E+00	53.7	53.0	0.00	100.0	0.0
5	5.00E−07	0.00E+00	0.00E+00	51.8	53.0	0.00	100.0	0.0
6	5.00E−07	0.00E+00	0.00E+00	92.7	95.8	0.00	100.0	0.0
7	5.00E−07	0.00E+00	0.00E+00	95.9	99.8	0.00	100.0	0.0
RMSE: 2.4%, $R^2 = 0.999$								
4	5.00E−07	1.20E−05	4.10E−06	42.5	44.3	0.00	99.41	0.59
4	5.00E−07	1.20E−05	4.10E−06	41.4	44.3	0.00	99.41	0.59
5	5.00E−07	3.90E−07	1.30E−07	58.2	54.2	0.00	100.00	0.00
5	5.00E−07	3.90E−07	1.30E−07	56.7	54.2	0.00	100.00	0.00
7	5.00E−07	7.60E−10	2.50E−10	91.9	99.8	0.00	100.00	0.00
7	5.00E−07	7.60E−10	2.50E−10	90.3	99.8	0.00	100.00	0.00
4	5.00E−07	3.90E−05	1.30E−05	53.6	63.3	0.00	97.17	2.83
5	5.00E−07	1.20E−06	4.10E−07	66.7	56.9	0.00	100.00	0.00
5	5.00E−07	1.20E−06	4.10E−07	65.1	56.9	0.00	100.00	0.00
6	5.00E−07	4.20E−08	1.40E−08	96.4	95.8	0.00	100.00	0.00
6	5.00E−07	4.20E−08	1.40E−08	98.5	95.8	0.00	100.00	0.00
7	5.00E−07	2.40E−09	8.00E−10	99.6	99.8	0.00	100.00	0.00
4	5.00E−07	1.20E−04	4.10E−05	61.3	72.1	0.00	93.67	6.33
5	5.00E−07	3.90E−06	1.30E−06	70.7	69.3	0.00	100.00	0.00
5	5.00E−07	3.90E−06	1.30E−06	72.9	69.3	0.00	100.00	0.00
6	5.00E−07	1.30E−07	4.40E−08	96.6	95.9	0.00	100.00	0.00
4	5.00E−07	1.20E−03	4.10E−04	85.9	86.9	20.96	61.72	17.32
5	5.00E−07	3.90E−05	1.30E−05	93.4	98.2	0.00	99.31	0.69
5	5.00E−07	3.90E−05	1.30E−05	96.1	98.2	0.00	99.31	0.69
7	5.00E−07	7.60E−08	2.50E−08	99.6	99.8	0.00	100.00	0.00
RMSE: 5.5%, $R^2 = 0.929$								

S8. Comparative Analysis of Pb and Other Metal Ions in the Formation of Ternary Complexes with PO_4 on Iron (Hydr)oxides

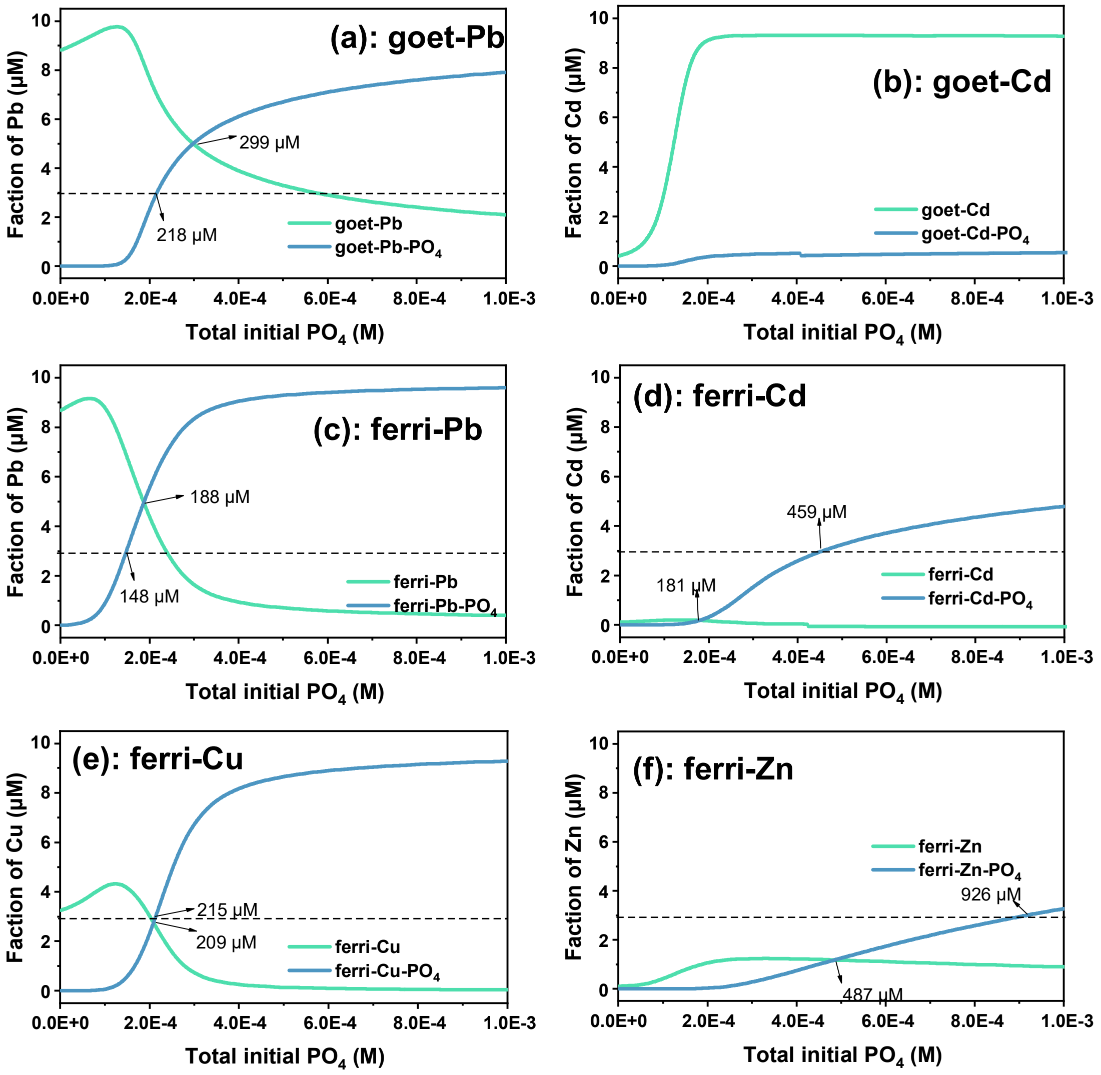

Figure S6. Comparison of the ability of Pb and other metal cations to form ternary metal-PO_4-mineral complexes on goethite and ferrihydrite based on the CD-MUSIC model. The total metal ion concentration is 10 μM, the background is 10 mM $NaNO_3$, the total concentration of iron minerals is 100 m^2/L, and the pH is 5.0 (equilibrium pH). (a), Pb-PO_4-goethite system, model parameters are from this study; (b), Cd-PO_4-goethite system, model parameters are from Deng et al., (2023); (c), Pb-PO_4-ferrihydrite system, model parameters are from Tiberg et al., (2013); (d), Cd-PO_4-ferrihydrite system, model parameters are from Tiberg and Gustafsson, (2016); (e), Cu-PO_4-ferrihydrite system, model parameters are from Tiberg et al., (2013); (f), Zn-PO_4-ferrihydrite system, model parameters are from Van Eynde et al., (2022). goet-Me represents the surface species of non-ternary complexes (green line), while goet-Me-PO_4 represents the surface species of ternary complexes (blue line). The intersection of

the green and blue lines is marked in the figure, representing the initial total PO_4 concentration when the contribution of ternary and non-ternary complexes to metal removal are equal. The intersection of the horizontal dashed line and the blue line indicates that a significant ternary complex has occurred, which is defined here as the corresponding initial total concentration of PO_4 when the ternary complex has removed 3 μM metal ion (accounting for 30% of the total metal concentration).

S9. Modeling Pb Immobilization on Goethite in the Presence of PO_4 Using Different Solubility Product of Hydropyromorphite

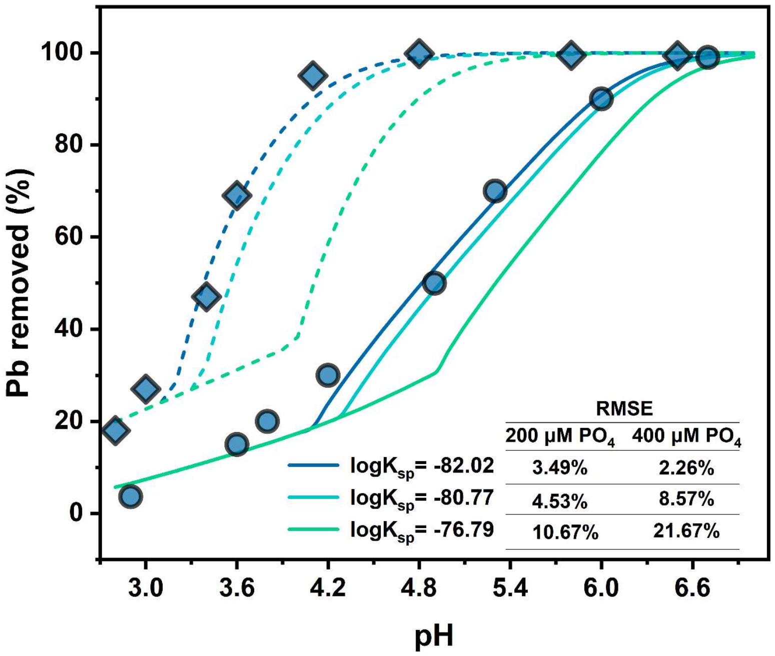

Figure S7. CD-MUSIC modeling (Model A) of Pb (300 μM) immobilization on goethite (1.3 g/L, 105 m^2/L) in the presence of 200 or 400 μM PO_4 (10 mM $NaNO_3$) using different solubility product (log K_{sp}) of HPM. Log K_{sp} value of −82.02, −80.77, and −76.79 are respectively from this study, Zhu et al., (2015), and Lindsay (1979). Symbols are experimental data, lines are CD-MUSIC modeling. Solid lines are 300 μM Pb with 200 μM PO_4, dashed lines are 300 μM Pb with 400 μM PO_4.

S10. Additional Results of Pb L3-Edge and P K-Edge XANES Spectra and LCF Analysis

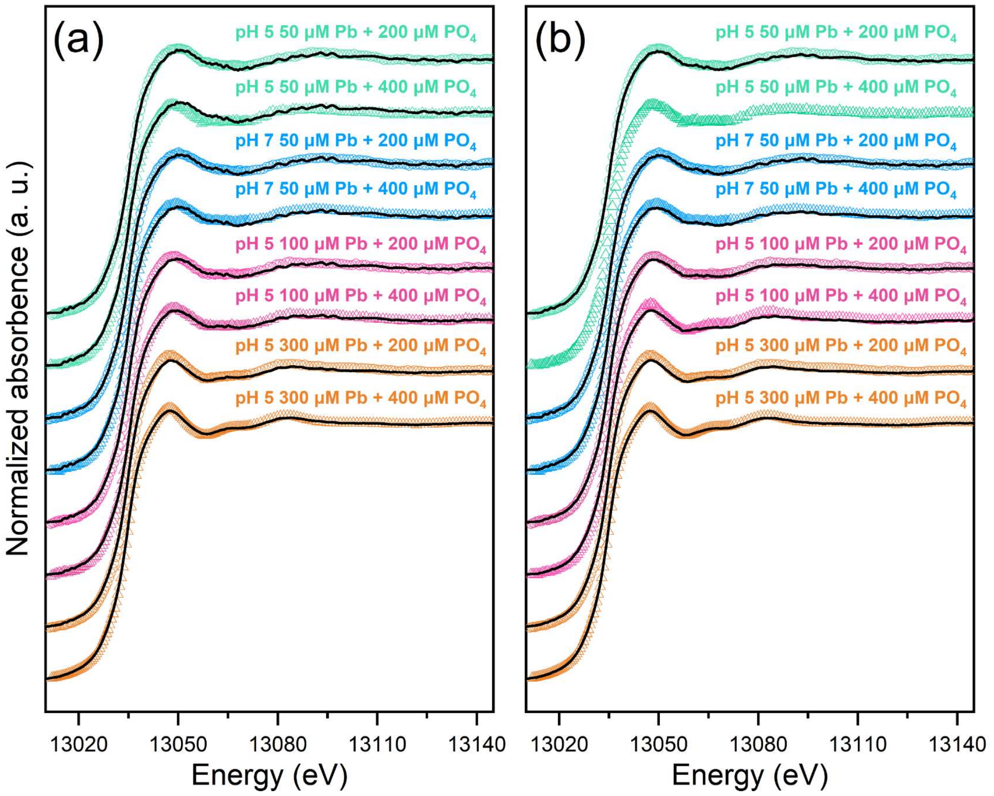

Figure S8. LCF analysis results of Pb L3-edge spectra without (a) and with (b) considering Pb-PO_4 ternary complex. Symbols are experimental results and black lines are LCF results. The quantitative results are presented in Table 2.

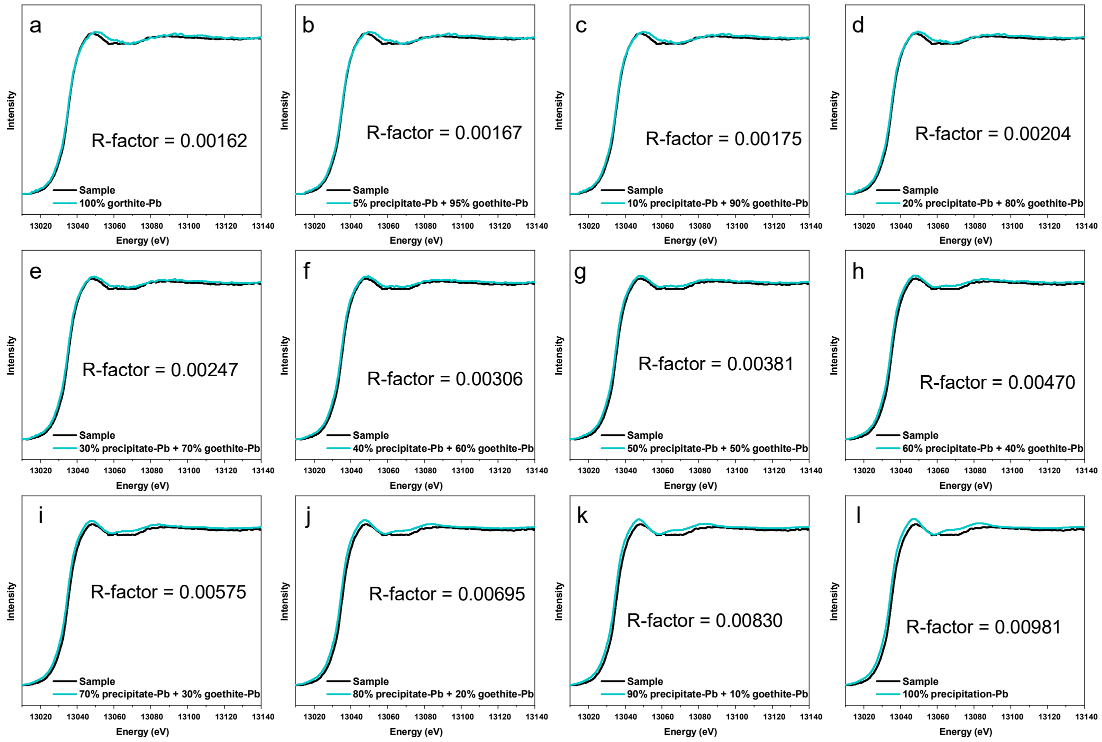

Figure S9. LCF fitting quality of Pb L3-edge spectra for the sample of 50 μM Pb + 400 μM PO_4 + goethite at pH 5, using different proportions of bidentate adsorbed Pb and precipitated Pb (HPM). R-factor = $\sum_i(\text{exp.} - \text{fit})^2/\sum_i(\text{exp.})^2$.

Table S5. Correlations and RMSEs between Pb surface species quantified by Pb L3-edge XANES-LCF analysis and CD-MUSIC modeling (both Model A and Model B).

XANES-LCF	CD-MUSIC modeling					
	Distinguish total adsorbed and precipitate				Ternary complex	
	Model A[a]		Model B[b]			
	R^2	RMSE (%)	R^2	RMSE (%)	R^2	RMSE (%)
Pb L3-edge fitting without ternary complex	0.957	6.5	0.870	15.0		
Pb L3-edge fitting with ternary complex	0.958	6.0	0.897	11.9	0.757	9.1

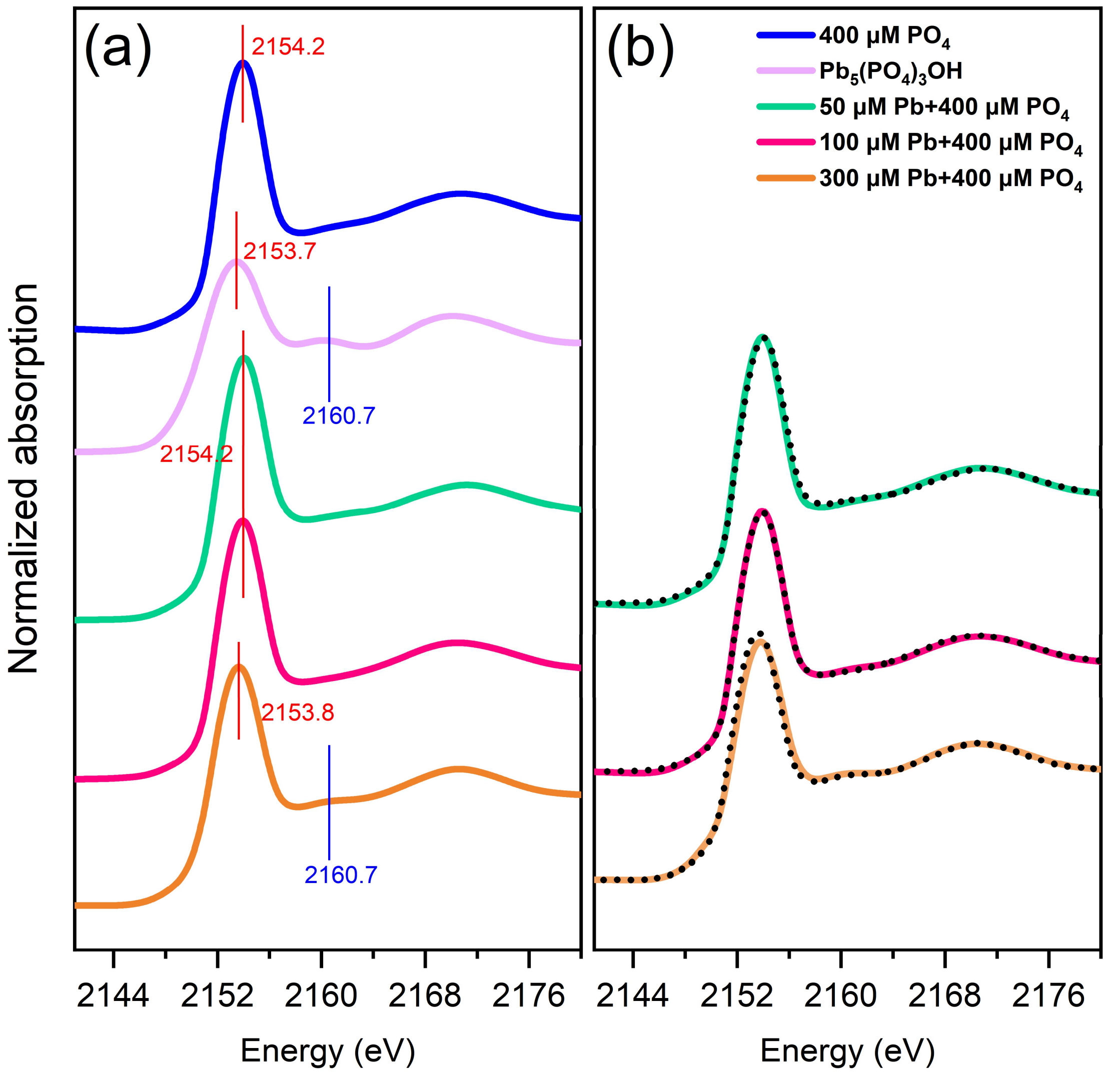

Figure S10. Normalized P K-edge XANES spectra of goethite with 400 μM PO_4 in the absence and presence of Pb at various concentrations at pH 5 in 10 mM $NaNO_3$ background, along with the XANES spectra of HPM (a) and the LCF analysis results (b). In (b), the black dashed lines are experimental data, while the colored solid lines are the LCF fitted values. The quantitative fitting results are shown in Table S6.

Table S6. Quantitative analysis of PO_4 species on goethite in the presence of Pb using LCF analysis of P K-edge XANES spectra and CD-MUSIC modeling (10 mM $NaNO_3$ background).

Samples	LCF analysis (%)				CD-MUSIC (%)				Precipitated Pb amount[d]	P: Pb in precipitate phase[e]	Calculated precipitated Pb (%)[f]
					Model A[b]		Model B[c]				
	Ads.	Pre.	R-factor[a]	χ^2	Ads.	Pre.	Ads.	Pre.			
pH 5 50 μM Pb + 400 μM PO_4 (63% PO_4 removed)	100(7.4)	0(7.4)	0.0030	0.0373	100	0	97.8	3.2	0	/	0
pH 5 100 μM Pb + 400 μM PO_4 (70% PO_4 removed)	92.3(0.8)	7.7(0.8)	0.0003	0.0040	91.7	8.3	86.0	14.0	39.4%/38.3%	0.588/0.605	36.8
pH 5 300 μM Pb + 400 μM PO_4 (94% PO_4 removed)	60.4(3.8)	39.6(3.8)	0.0077	0.0872	61.4	38.6	57.9	42.1	85.1%/82.0%	0.597/0.619	80.2

a. Parameter of the goodness of fit. R-factor=$\sum_i(\text{Exp.} - \text{Fit})^2/\sum_i(\text{Exp.})^2$, where Exp. means experimental value of XANES, Fit means LCF results;

b. and c. CD-MUSIC calculations with and without considering Pb-PO_4 ternary complexes. Ads: adsorbed. Pre: precipitated. The total ads. in Model A are the sum of monodentate and bidentate bound PO_4 as well as PO_4 in the Pb-PO_4 ternary complex on goethite. For further details, please refer to S3 of SI;

d. Precipitate Pb analyzed by Pb-L3 edge XANES-LCF using two/three end members, respectively.

e. Atomic ratios of P to Pb in the sample based on LCF analysis results of Pb L3-edge (two/three end members) and P K-edge;

f. Calculated amount of precipitated Pb based on the LCF analysis results of P K-edge and the atomic ratio of P: Pb of 0.6 in the precipitation phase.

S11. Quantifying Contribution of Different Mechanisms to Pb Immobilization on Goethite Induced by Phosphate Using the CD-MUSIC Model

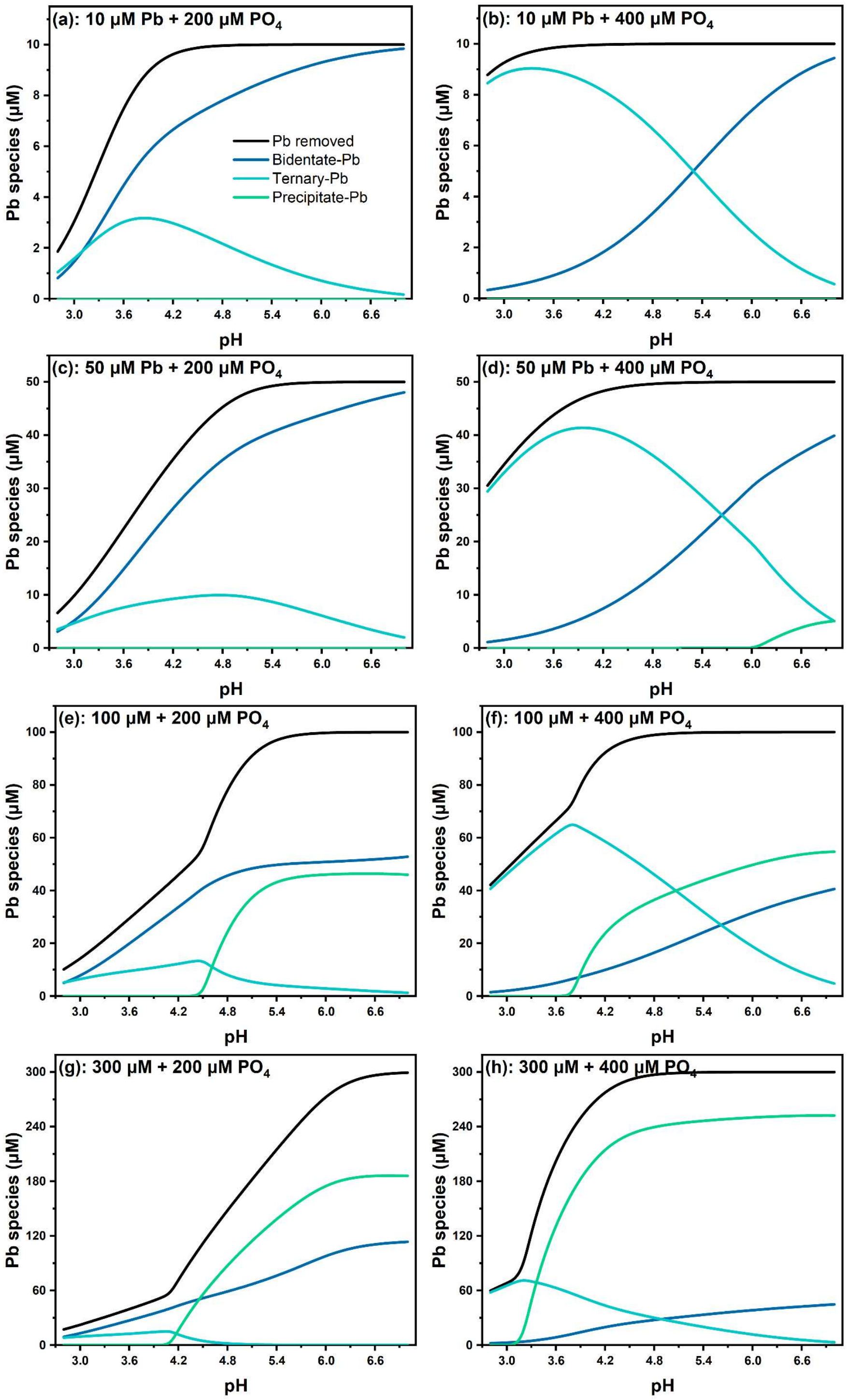

Figure S11. Contribution of different mechanisms to PO_4 induced immobilization of Pb on goethite as a function of pH. Blue lines: bidentate complexation; teal lines: ternary complexation; green lines: HPM precipitation. The CD-MUSIC parameters used are listed in Table 1. Goethite: 1.3 g/L (105 m^2/L). Background: 10 mM $NaNO_3$.

S12. Additional Results of Pb L3-Edge EXAFS Analysis

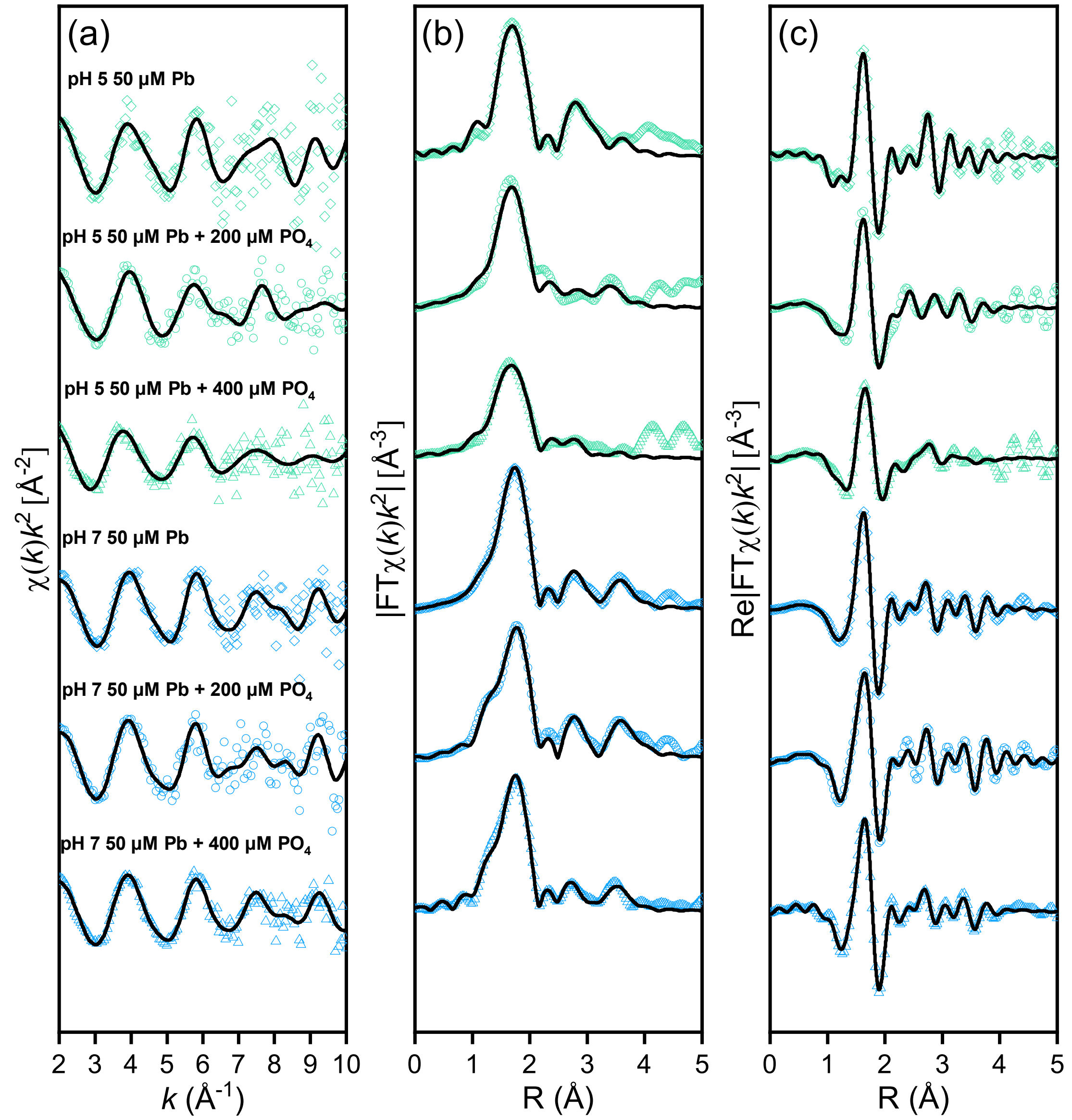

Figure S12. Normalized k^2-weighted experimental Pb L3-edge EXAFS spectra and fitted value (a), with corresponding Fourier transformed magnitude (b) and real parts (c) of Fourier transform. Fitting parameters are listed in Table 3. The details in FEFF fitting of EXAFS are provided in S3 of SI.

Table S7. EXAFS results of Pb complex with goethite in previous studies.

Samples	Pb–O		Pb–Fe		Pb–Fe		Ref.
	CN	Length (Å)	CN	Length (Å)	CN	Length (Å)	
**pH 5** 450 μM Pb + 5 g/L goethite	2.3(1)	2.28(1)	0.4(1)	3.34(3)	0.4(2)	3.88(3)	Ostergren et al., (1999)
**pH 5** 100 μM Pb + 0.4 g/L goethite	2.5	2.29	0.8	3.36	0.6	3.93	Elzinga et al., (2001)
**pH 6** 1190 μM Pb + 5.3 g/L goethite	2.1(8)	2.33(1)	0.3(1)	3.36(3)	0.3(13)	3.92(4)	Bargar et al., (1998)
**pH 7** 240 μM Pb + 2.6 g/L goethite	2.5(1)	2.29(1)	0.5(1)	3.34(2)	0.6(14)	3.93(3)	
**N. A.**	1.8(1)	2.31(1)	0.3(2)	3.31(3)			Liu et al., (2018)
**pH 5** 50 μM Pb + 1.3 g/L goethite	1.8(3)	2.28(1)	0.8(2)	3.37(1)	0.4(3)	4.02(5)	This study
**pH 7** 50 μM Pb + 1.3 g/L goethite	2.0(1)	2.29(1)	0.5(1)	3.35(1)	0.8(1)	4.01(1)	

Table S8. Coordination environment parameters of EXAFS results fitted by FEFF using Pb–Fe path instead of Pb–P path.

Sample	Path	CN^a	R^b (Å)	σ^{2c} ($Å^2$)	$\Delta E_0{}^d$ (eV)	R-factor[e]
pH 5	Pb–O	1.9(4)	2.27(1)	0.006(3)	−9.9	0.078
50 μM Pb+200 μM PO_4	Pb–Fe	0.3(4)	3.51(9)			
	Pb–Fe	0.4(6)	3.91(4)			
pH 5	Pb–O	2.5(4)	2.32(1)	0.013(3)	−9.9	0.044
50 μM Pb+400 μM PO_4	Pb–Fe	0.9(7)	3.79(6)			
	Pb–Fe	0.8(3)	3.97(6)			

a. Coordination number; b. Interatomic distance; c. Debye-Waller factor; d. Energy shift threshold; e. Goodness-of-fit parameter: The quality of the fit was assessed using the R-factor, calculated as $\Sigma(\chi_{data} - \chi_{fit})^2/\Sigma(\chi_{data})^2$, where χ_{data} and χ_{fit} represent the experimental and calculated structure factors, respectively. A value of R-factor below 0.05 indicates a good fit quality; The passive amplitude reduction factor ($S_0{}^2$) for all samples was set to 0.8. The estimated parameter uncertainties are presented in parentheses, indicating the magnitude of the errors associated with the last significant digit of the reported values. The details in FEFF fitting of EXAFS are provided in S3 of SI.

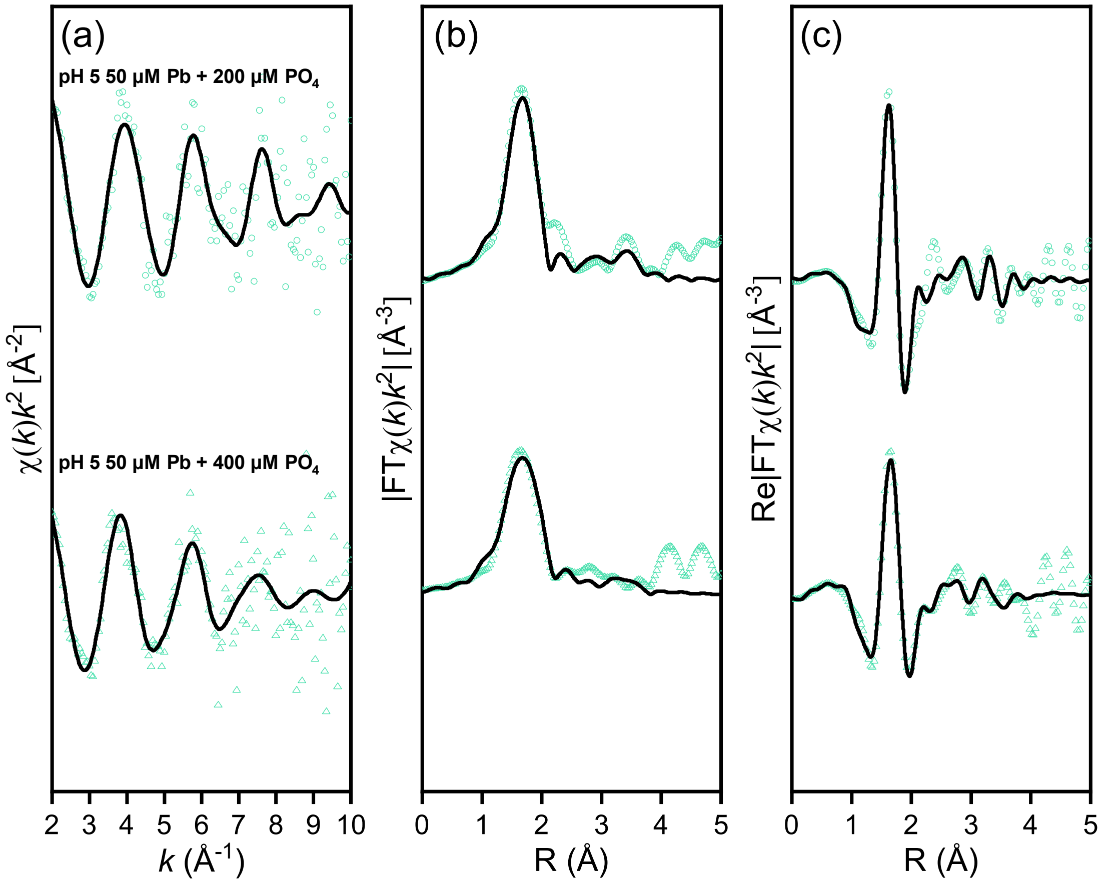

Figure S13. Normalized k^2-weighted experimental Pb L3-edge EXAFS spectra and fitted value by using Pb–Fe path instead of Pb–P path as the second shell (a), with corresponding Fourier transformed magnitude (b) and real parts (c) of Fourier transform. The details in FEFF fitting of EXAFS are provided in S3 of SI.

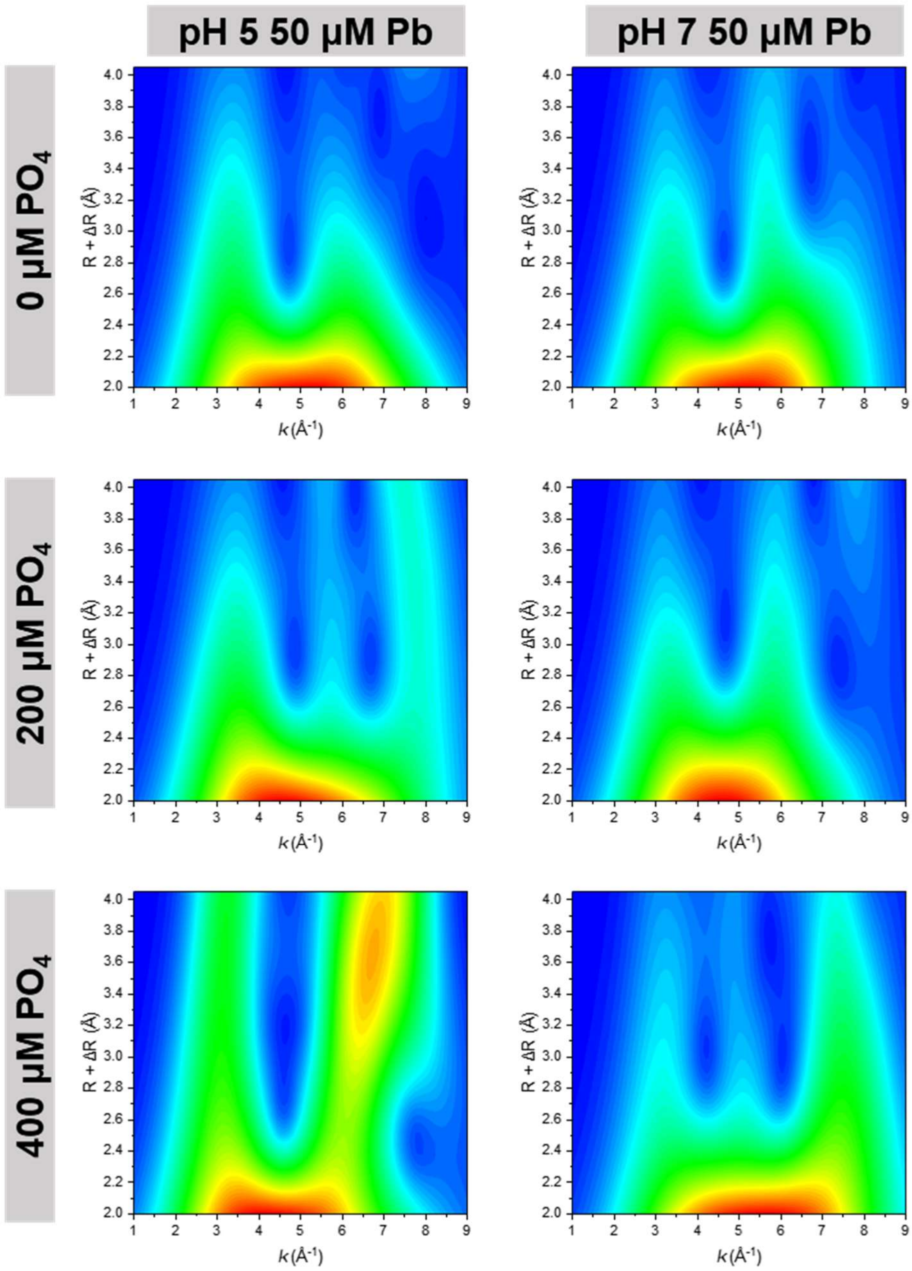

Figure S14. Results from wavelet transform (WT) analysis of Pb EXAFS spectra. *k*-range used was 3–8 Å^{-1} for all samples. X-axis (*k*) indicates energy expressed as the photoelectron wavenumber and y-axis (*R*) is not corrected for phase shift. Red/yellow areas indicate high intensity whereas green/blue areas indicate low intensity of the WT modulus.

S13. Analysis of Surface Charge Properties Using CD-MUSIC Model and DFT Calculations

Table S9. Electrostatic potential area from ESP analysis and charge distribution from BVC analysis based on the DFT calculation above the Fe-cluster of the interested surface complexes. APEP: area of positive electrostatic potential; ANEP: area of negative electrostatic potential. The BVC analysis results regard Pb or P atoms directly complexed with ≡$FeOH^{-0.5}$.

Surface complex	ESP analysis			BVC analysis				
	Atom/ H_2O	APEP ($Å^2$)	ANEP ($Å^2$)	Bond	*R* (pm)	*v* (v. u)	Δz_0	Δz_1
Lead bidentate	Pb1	19.41	0.00	FeOH–Pb	232.1	0.66	1.28	0.72
	H_2O–1	20.98	0.00	FeOH–Pb	231.0	0.68		
	H_2O–2	28.74	0.00	Pb–H_2O1	250.0	0.41		
	H_2O–3	31.93	0.00	Pb–H_2O2	270.1	0.24		
	H_2O–4	27.86	0.00	Pb–H_2O3	376.2	0.01		
	Sum	128.92	0.00	Pb–H_2O4	440.0	0.00		
	APEP – ANEP = 128.92			R_o[a] = 216.74 pm				
Phosphate bidentate	P1	0.00	0.20	FeO–P	161.5	1.10	0.34	−1.34
	O1	0.00	27.25	FeO–P	161.5	1.10		
	O2	0.00	23.29	P–O	152.6	1.40		
	Sum	0.00	50.74	P–O	152.7	1.40		
	APEP – ANEP = −50.74			R_o = 165.10 pm				
Phosphate monodentate	P1	0.00	0.32	FeO–P	156.8	1.25	0.29	−1.29
	O1	0.00	25.81	P–O	150.6	1.48		
	O2	0.00	16.10	P–O	153.8	1.36		
	O3	0.00	6.42	P–OH	168.6	0.91		
	H1	0.00	14.55					
	Sum	0.00	63.20					
	APEP – ANEP = −63.20			R_o = 165.11 pm				
Lead bridged ternary	Pb1	17.25	0.00	FeOH–Pb	231.0	0.65	1.22	−0.22
	P1	0.10	0.00	FeOH–Pb	232.7	0.62		
	O1	14.54	0.00	Pb–OP	236.1	0.56		
	O2	1.02	13.96	Pb–H_2O1	391.3	0.01		
	O3	2.45	11.43	Pb–H_2O2	283.5	0.16		
	O4	9.38	2.73	Pb–H_2O3	436.2	0.00		
	H1	14.71	0.00					
	H2	3.40	0.00					
	H_2O–1	26.26	0.00					
	H_2O–2	27.97	0.00					
	H_2O–3	22.77	0.69					

	Sum	139.84	28.80					
	APEP – ANEP = 111.04			R_o = 224.9 pm				
Phosphate bridged ternary	Pb1	14.76	0.00	FeO–P	158.0	1.20	0.57	0.43
	P1	0.07	0.00	FeO–P	155.4	1.28		
	O1	19.01	0.44	P–OPb	157.4	1.22		
	O2	1.41	0.00	P–OPb	154.9	1.30		
	H_2O–1	33.22	0.00					
	H_2O–2	24.25	0.00					
	H_2O–3	32.20	0.00					
	H_2O–4	18.33	0.00					
	Sum	143.24	0.44					
	APEP – ANEP = 142.80			R_o = 164.81 pm				
Monodentate -O-sharing ternary	Pb1	13.28	0.00	Centered on Pb; $R_{o,Pb}$ = 211.5 pm				
	P1	0.03	0.00	FeOH–Pb	230.5	0.67	0.64	1.36
	O1	8.33	0.00	Pb–H_2O1	297.0	0.11		
	O2	7.71	1.50	Pb–H_2O2	287.6	0.14		
	O3	13.81	0.00	Pb–H_2O3	243.1	0.47		
	H1	14.92	0.00	Pb–H_2O4	403.6	0.01		
	H_2O–1	19.50	0.00	Pb–OP	234.6	0.60		
	H_2O–2	29.46	0.00					
	H_2O–3	32.51	0.00	Centered on P; $R_{o,P}$ = 164.6 pm				
	H_2O–4	13.28	0.00	FeO–P	154.6	1.31	0.35	−1.35
				P–OH	162.4	1.06		
				P–O	153.0	1.37		
				P–OPb	156.2	1.26		
	Sum	139.54	1.50	sum			0.99	0.01
	APEP – ANEP = 138.04							
Phosphate protonated bidentate [b]	APEP – ANEP = −5.33<br>Δz_0 = 0.65; Δz_1 = −0.65 in CD-MUSIC model			FeO–P	154.3	1.33	0.47	−0.47
				FeO–P	163.6	1.03		
				P–O	151.3	1.44		
				P–OH	157.8	1.21		
				R_o = 164.7 pm				

a. R_o (pm) is a reference distance whose value is chosen in such a way that the sum of v is equal to the formal valence of the adsorbing ion. b. Cited from Ma et al., (2023), detailed bond length was obtained by personal communication. For more details regarding ESP and BVC analysis, please refer to S4 of SI.

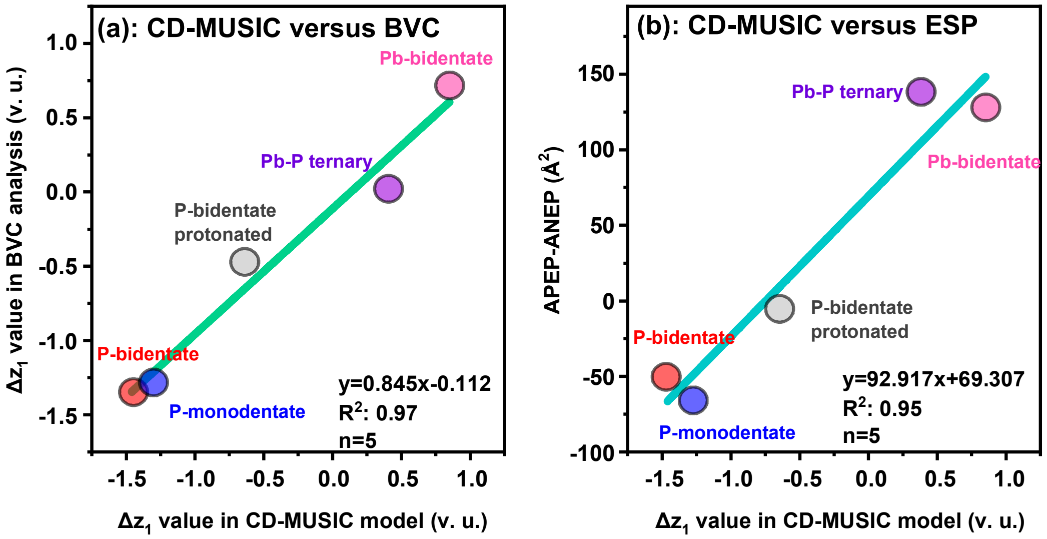

Figure S15. Correlation of charge distribution properties derived from DFT calculations and from CD-MUSIC model. (a). Correlation between Δz_1 values of surface complexes between CD-MUSIC model and the bond valence concept (BVC). (b). Correlation between Δz_1 values of surface complexes from CD-MUSIC model and their corresponding APEP − ANEP (difference in the area of positive and negative potential from ESP analysis). In addition to data of current study, a protonated bidentate PO_4 species on iron clusters was included (grey symbols), with its Δz_1 value from Hiemstra and Zhao (2016) and bond lengths (for BVC analysis) and APEP − ANEP values from Ma et al. (2023). "Pb-P ternary" represents the monodentate-oxygen-sharing ternary structure. The detailed results of ESP and BVC analysis are provided in Table S9.

Reference.

Adamo, C., Barone, V., 1999. Toward reliable density functional methods without adjustable parameters: The PBE0 model. J. Chem. Phys. 110, 6158–6170. https://doi.org/10.1063/1.478522

Antelo, J., Arce, F., Fiol, S., 2015. Arsenate and phosphate adsorption on ferrihydrite nanoparticles. synergetic interaction with calcium ions. Chem. Geol. 410, 53–62. https://doi.org/10.1016/j.chemgeo.2015.06.011

Bargar, J.R., Brown, G.E., Parks, G.A., 1998. Surface complexation of Pb(II) at oxide-water interfaces: III. XAFS determination of Pb(II) and Pb(II)-chloro adsorption complexes on goethite and alumina. Geochim. Cosmochim. Acta 62, 193–207. https://doi.org/10.1016/s0016-7037(97)00334-7

Barinova, A., Bonin, M., Pushcharovskii, D., Rastsvetaeva, R., Schenk, K., Dimitrova, O., 1998. Crystal structure of synthetic hydroxylpyromorphite $Pb_5(PO_4)_3(OH)$. Crystallogr. Rep. 43, 189–192.

Blöchl, P.E., 1994. Projector augmented-wave method. Phys. Rev. B 50, 17953–17979. https://doi.org/10.1103/PhysRevB.50.17953

Brown, I.D., Altermatt, D., 1985. Bond-valence parameters obtained from a systematic analysis of the inorganic crystal structure database. Acta Crystallogr. B 41, 244–247. https://doi.org/10.1107/S0108768185002063

Deng, Y., Ren, C., Chen, N., Huang, Y., Zhu, G., Zhang, X., Weng, L., Li, Y., 2023. Effects of pH and phosphate on cadmium adsorption onto goethite and a paddy soil: Experiments and NOM-CD model. J. Soils Sediments 1–11. https://doi.org/10.1007/s11368-023-03481-3

Dohm, S., Hansen, A., Steinmetz, M., Grimme, S., Checinski, M.P., 2018. Comprehensive thermochemical benchmark set of realistic closed-shell metal organic reactions. J. Chem. Theory Comput. 14, 2596–2608. https://doi.org/10.1021/acs.jctc.7b01183

Elzinga, E.J., Peak, D., Sparks, D.L., 2001. Spectroscopic studies of Pb(II)-sulfate interactions at the goethite-water interface. Geochim. Cosmochim. Acta 65, 2219–2230. https://doi.org/10.1016/S0016-7037(01)00595-6

Fabiano, E., Constantin, L.A., Della Sala, F., 2010. Generalized gradient approximation bridging the rapidly and slowly varying density regimes: A PBE-like functional for hybrid interfaces. Phys. Rev. B 82, 113104. https://doi.org/10.1103/PhysRevB.82.113104

Frisch, M. ea, Trucks, G.W., Schlegel, H.B., Scuseria, G.E., Robb, Ma., Cheeseman, J.R., Scalmani, G., Barone, V., Petersson, G.A., Nakatsuji, H., 2016. Gaussian 16. Gaussian, Inc. Wallingford, CT.

Fuente, S.A., Belelli, P.G., Castellani, N.J., Avena, M., 2013. LDA + *U* and GGA + *U* studies of Al-rich and bulk goethite (α-FeOOH). Mater. Chem. Phys. 137, 1012–1020. https://doi.org/10.1016/j.matchemphys.2012.11.017

Goli, E., Rahnemaie, R., Hiemstra, T., Malakouti, M.J., 2011. The interaction of boron with goethite: Experiments and CD–MUSIC modeling. Chemosphere 82, 1475–1481. https://doi.org/10.1016/j.chemosphere.2010.11.034

Grimme, S., Antony, J., Ehrlich, S., Krieg, H., 2010. A consistent and accurate ab initio parametrization of density functional dispersion correction (DFT-D) for the 94 elements H-pu. J. Chem. Phys. 132, 154104. https://doi.org/10.1063/1.3382344

Hafner, J., 2008. Ab-initio simulations of materials using VASP: Density-functional theory and beyond. J. Comput. Chem. 29, 2044–2078.

Hariharan, P.C., Pople, J.A., 1973. The influence of polarization functions on molecular orbital hydrogenation energies. Theor. Chim. Acta 28, 213–222. https://doi.org/10.1007/BF00533485

Hiemstra, T., Barnett, M.O., van Riemsdijk, W.H., 2007. Interaction of silicic acid with goethite. J. Colloid Interface Sci. 310, 8–17. https://doi.org/10.1016/j.jcis.2007.01.065

Hiemstra, T., Van Riemsdijk, W.H., 2006. On the relationship between charge distribution, surface hydration, and the structure of the interface of metal hydroxides. J. Colloid Interface Sci. 301, 1–18. https://doi.org/10.1016/j.jcis.2006.05.008

Hiemstra, T., Van Riemsdijk, W.H., 1999. Surface structural ion adsorption modeling of competitive binding of oxyanions by metal (hydr)oxides. J. Colloid Interface Sci. 210, 182–193. https://doi.org/10.1006/jcis.1998.5904

Hiemstra, T., Zhao, W., 2016. Reactivity of ferrihydrite and ferritin in relation to surface structure, size, and nanoparticle formation studied for phosphate and arsenate. Environ. Sci. Nano 3, 1265–1279. https://doi.org/10.1039/C6EN00061D

Hohenberg, P., Kohn, W., 1964. Inhomogeneous electron gas. Phys. Rev. 136, B864–B871. https://doi.org/10.1103/PhysRev.136.B864

Humphrey, W., Dalke, A., Schulten, K., 1996. VMD: Visual molecular dynamics. J. Mol. Graph. 14, 33–38. https://doi.org/10.1016/0263-7855(96)00018-5

Karthikeyan, K.G., Elliott, H.A., 1999. Surface complexation modeling of copper sorption by hydrous oxides of iron and aluminum. J. Colloid Interface Sci. 220, 88–95. https://doi.org/10.1006/jcis.1999.6507

Kohn, W., Sham, L.J., 1965. Self-consistent equations including exchange and correlation effects. Phys. Rev. 140, A1133–A1138. https://doi.org/10.1103/PhysRev.140.A1133

Kresse, G., Furthmüller, J., 1996. Efficient iterative schemes for ab initio total-energy calculations using a plane-wave basis set. Phys. Rev. B 54, 11169–11186. https://doi.org/10.1103/PhysRevB.54.11169

Leciejewicz, J., 1961. On the crystal structure of tetragonal (red) PbO. Acta Crystallogr. 14, 1304–1304. https://doi.org/10.1107/S0365110X61003892

Leung, K., Criscenti, L.J., 2017. Lead and selenite adsorption at water–goethite interfaces from first principles. J. Phys. Condens. Matter 29, 365101. https://doi.org/10.1088/1361-648X/aa7e4f

Liang, Y., Yu, D., Jin, J., Xiong, J., Hou, J., Wang, M., Tan, W., 2021. Microstructure of Al-substituted goethite and its adsorption performance for Pb(II) and As(V). Sci. Total Environ. 790, 148202. https://doi.org/10.1016/j.scitotenv.2021.148202

Lindsay, W.L., 1979. Chemical equilibria in soils. Wiley, New York.

Liu, H., Lu, X., Li, M., Pan, C., Zhang, L., Zhang, R., Li, J., Xiang, W., 2018. Structural incorporation of manganese into goethite and its enhancement of Pb(II) adsorption. Environ. Sci. Technol. 52, 4719–4727. https://doi.org/10.1021/acs.est.7b05612

Lu, T., Chen, F., 2012. Multiwfn: A multifunctional wavefunction analyzer. J. Comput. Chem. 33, 580–592. https://doi.org/10.1002/jcc.22885

Lützenkirchen, J., Behra, Ph., 1995. On the surface precipitation model for cation sorption at the (hydr)oxide water interface. Aquat. Geochem. 1, 375–397. https://doi.org/10.1007/BF00702740

Ma, J., Li, J., Weng, L., Ouyang, X., Chen, Y., Li, Y., 2023. Phosphorus-enhanced and calcium-retarded transport of ferrihydrite colloid: Mechanism of electrostatic potential changes regulated via adsorption speciation. Environ. Sci. Technol. 57, 4219–4230. https://doi.org/10.1021/acs.est.2c09670

Manecki, M., Kwaśniak-Kominek, M., Majka, J.M., Rakovan, J., 2020. Model of interface-coupled dissolution-precipitation mechanism of pseudomorphic replacement reaction in aqueous solutions based on the system of cerussite $PbCO_3$ – pyromorphite $Pb_5(PO_4)_3Cl$. Geochim. Cosmochim. Acta 289, 1–13. https://doi.org/10.1016/j.gca.2020.08.015

Marenich, A.V., Cramer, C.J., Truhlar, D.G., 2009. Universal solvation model based on solute electron density and on a continuum model of the solvent defined by the bulk dielectric constant and atomic surface tensions. J. Phys. Chem. B 113, 6378–6396. https://doi.org/10.1021/jp810292n

Maurer, L.R., Bursch, M., Grimme, S., Hansen, A., 2021. Assessing density functional theory for chemically relevant open-shell transition metal reactions. J. Chem. Theory Comput. 17, 6134–6151. https://doi.org/10.1021/acs.jctc.1c00659

Mendez, J.C., Hiemstra, T., 2020. Ternary complex formation of phosphate with Ca and Mg ions binding to ferrihydrite: Experiments and mechanisms. ACS Earth Space Chem. 4, 545–557. https://doi.org/10.1021/acsearthspacechem.9b00320

Momma, K., Izumi, F., 2011. VESTA 3 for three-dimensional visualization of crystal, volumetric and morphology data. J. Appl. Crystallogr. 44, 1272–1276. https://doi.org/10.1107/S0021889811038970

Ostergren, J.D., Bargar, J.R., Brown, G.E., Parks, G.A., 1999. Combined EXAFS and FTIR investigation of sulfate and carbonate effects on Pb(II) sorption to goethite (α-FeOOH). J. Synchrotron Radiat. 6, 645–647. https://doi.org/10.1107/S0909049598017750

Ostergren, J.D., Brown, G.E., Parks, G.A., Persson, P., 2000a. Inorganic ligand effects on Pb(II) sorption to goethite (α-FeOOH): II. sulfate. J. Colloid Interface Sci. 225, 483–493. https://doi.org/10.1006/jcis.1999.6702

Ostergren, J.D., Trainor, T.P., Bargar, J.R., Brown, G.E., Parks, G.A., 2000b. Inorganic ligand effects on Pb(II) sorption to goethite (α-FeOOH): I. carbonate. J. Colloid Interface Sci. 225, 466–482. https://doi.org/10.1006/jcis.1999.6701

Paul, K.W., Kubicki, J.D., Sparks, D.L., 2007. Sulphate adsorption at the Fe (hydr)oxide–H_2O interface: Comparison of cluster and periodic slab DFT predictions. Eur. J. Soil Sci. 58, 978–988. https://doi.org/10.1111/j.1365-2389.2007.00936.x

Perdew, J.P., Burke, K., Ernzerhof, M., 1996. Generalized gradient approximation made simple. Phys. Rev. Lett. 77, 3865–3868. https://doi.org/10.1103/PhysRevLett.77.3865

Rahnemaie, R., Hiemstra, T., van Riemsdijk, W.H., 2007a. Geometry, charge distribution, and surface speciation of phosphate on goethite. Langmuir 23, 3680–3689. https://doi.org/10.1021/la062965n

Rahnemaie, R., Hiemstra, T., van Riemsdijk, W.H., 2007b. Carbonate adsorption on goethite in competition with phosphate. J. Colloid Interface Sci. 315, 415–425. https://doi.org/10.1016/j.jcis.2007.07.017

Ravel, B., Newville, M., 2005. ATHENA, ARTEMIS, HEPHAESTUS: Data analysis for X-ray Absorption Spectroscopy using IFEFFIT. J. Synchrotron Radiat. 12, 537–541. https://doi.org/10.1107/S0909049505012719

Schwerdtfeger, P., Dolg, M., Schwarz, W.H.E., Bowmaker, G.A., Boyd, P.D.W., 1989. Relativistic effects in gold chemistry. I. Diatomic gold compounds. J. Chem. Phys. 91, 1762–1774. https://doi.org/10.1063/1.457082

Tiberg, C., Gustafsson, J.P., 2016. Phosphate effects on cadmium(II) sorption to ferrihydrite. J. Colloid Interface Sci. 471, 103–111. https://doi.org/10.1016/j.jcis.2016.03.016

Tiberg, C., Sjöstedt, C., Persson, I., Gustafsson, J.P., 2013. Phosphate effects on copper(II) and lead(II) sorption to ferrihydrite. Geochim. Cosmochim. Acta 120, 140–157. https://doi.org/10.1016/j.gca.2013.06.012

Van Eynde, E., Hiemstra, T., Comans, R.N.J., 2022. Interaction of Zn with ferrihydrite and its cooperative binding in the presence of PO_4. Geochim. Cosmochim. Acta, 320, 223–237. https://doi.org/10.1016/j.gca.2022.01.010

Venema, P., Hiemstra, T., van Riemsdijk, W.H., 1996. Multisite adsorption of cadmium on goethite. J. Colloid Interface Sci. 183, 515–527. https://doi.org/10.1006/jcis.1996.0575

Villalobos, M., Pérez-Gallegos, A., 2008. Goethite surface reactivity: A macroscopic investigation unifying proton, chromate, carbonate, and lead(II) adsorption. J. Colloid Interface Sci. 326, 307–323. https://doi.org/10.1016/j.jcis.2008.06.026

Wang, X., Zhang, Y., Song, C., Shen, Z., Wang, T., Yang, K., Miao, H., Yang, J., Wang, J., Xu, X., 2024. Novel insight into the competitive adsorption behaviors of As(V), Sb(V), and P(V) on {110} facets of goethite: Existing form and coordination structure affinity. Chem. Eng. J. 479, 147677. https://doi.org/10.1016/j.cej.2023.147677

Weng, L., Temminghoff, E.J., Van Riemsdijk, W.H., 2001. Contribution of individual sorbents to the control of heavy metal activity in sandy soil. Environ. Sci. Technol. 35, 4436–4443. https://doi.org/10.1021/es010085j

Wu, J., Zhao, X., Li, Z., Gu, X., 2020. Thermodynamic and kinetic coupling model of Cd(II) and Pb(II) adsorption and desorption on goethite. Sci. Total Environ. 727, 138730. https://doi.org/10.1016/j.scitotenv.2020.138730

Xie, L., Giammar, D.E., 2007. Chapter 13 influence of phosphate on adsorption and surface precipitation of lead on iron oxide surfaces, in: Barnett, M.O., Kent, D.B. (Eds.), Developments in Earth and Environmental Sciences, Adsorption of Metals

by Geomedia II: Variables, Mechanisms, and Model Applications. Elsevier, pp. 349–373. https://doi.org/10.1016/S1571-9197(07)07013-9
Xu, J., Gu, X., Guo, Y., Tong, F., Chen, L., 2016. Adsorption behavior and mechanism of glufosinate onto goethite. Sci. Total Environ. 560–561, 123–130. https://doi.org/10.1016/j.scitotenv.2016.03.239
Yan, W., Jing, C., 2018. Molecular insights into glyphosate adsorption to goethite gained from ATR-FTIR, two-dimensional correlation spectroscopy, and DFT study. Environ. Sci. Technol. 52, 1946–1953. https://doi.org/10.1021/acs.est.7b05643
Zhihang, 2023. WtEXAFS. https://github.com/Himmelspol/wtEXAFS. Accessed at 2024. 06. 05.
Zhu, Y., Zhu, Z., Zhao, X., Liang, Y., Huang, Y., 2015. Characterization, dissolution, and solubility of lead hydroxypyromorphite [$Pb_5(PO_4)_3OH$] at 25–45°C. J. Chem. 2015, e269387. https://doi.org/10.1155/2015/269387
